# Expansion microscopy of *Plasmodium* gametocytes reveals the molecular architecture of a bipartite microtubule organisation centre coordinating mitosis with axoneme assembly

**DOI:** 10.1371/journal.ppat.1010223

**Published:** 2022-01-25

**Authors:** Ravish Rashpa, Mathieu Brochet

**Affiliations:** University of Geneva, Department of Microbiology and Molecular Medicine, Faculty of Medicine, Geneva, Switzerland; Loyola University Chicago, UNITED STATES

## Abstract

Transmission of malaria-causing parasites to mosquitoes relies on the production of gametocyte stages and their development into gametes. These stages display various microtubule cytoskeletons and the architecture of the corresponding microtubule organisation centres (MTOC) remains elusive. Combining ultrastructure expansion microscopy (U-ExM) with bulk proteome labelling, we first reconstructed in 3D the subpellicular microtubule network which confers cell rigidity to *Plasmodium falciparum* gametocytes. Upon activation, as the microgametocyte undergoes three rounds of endomitosis, it also assembles axonemes to form eight flagellated microgametes. U-ExM combined with Pan-ExM further revealed the molecular architecture of the bipartite MTOC coordinating mitosis with axoneme formation. This MTOC spans the nuclear membrane linking cytoplasmic basal bodies to intranuclear bodies by proteinaceous filaments. In *P*. *berghei*, the eight basal bodies are concomitantly *de novo* assembled in a SAS6- and SAS4-dependent manner from a deuterosome-like structure, where centrin, γ-tubulin, SAS4 and SAS6 form distinct subdomains. Basal bodies display a fusion of the proximal and central cores where centrin and SAS6 are surrounded by a SAS4-toroid in the lumen of the microtubule wall. Sequential nucleation of axonemes and mitotic spindles is associated with a dynamic movement of γ-tubulin from the basal bodies to the intranuclear bodies. This dynamic architecture relies on two non-canonical regulators, the calcium-dependent protein kinase 4 and the serine/arginine-protein kinase 1. Altogether, these results provide insights into the molecular organisation of a bipartite MTOC that may reflect a functional transition of a basal body to coordinate axoneme assembly with mitosis.

## Author summary

Malaria parasites rely on multiple phases of proliferation to build large populations from a small number of founding members, both in the human and the mosquito hosts. Malaria transmission relies on the production of gametes that follows ingestion by a mosquito. In particular, the *Plasmodium* microgametocyte rapidly forms eight flagellated microgametes through an unusual coupling of mitosis and axoneme formation via a single microtubule organization centre (MTOC). Due to evolutionarily divergence of *Plasmodium* parasites from well-studied organisms and their relatively small size, little is known about the molecular organisation and the dynamics of this dual MTOC. In this study, we have implemented expansion microscopy (ExM) techniques to image microtubule structures in *Plasmodium* gametocytes. ExM consists in physically expanding the cell while keeping its cellular structures intact to allow nanoscale imaging of fluorescently labelled structures with conventional microscopes. This reveals the highly dynamic molecular organisation of this bipartite MTOC regulated by two non-canonical kinase regulators as well as the requirement of conserved MTOC components to coordinate mitosis and axoneme assembly. Altogether our results allow to better understand how malaria-causing parasites replicate to form large populations and how they form flagella to colonise the mosquito.

## Introduction

Malaria is caused by intracellular parasites of the *Plasmodium* genus that are transmitted via the bite of an infected Anopheles mosquito. The major pathophysiological processes in malaria are linked to the proliferation of asexual blood stages, whereas transmission to the mosquito is solely initiated by an obligatory sexual life cycle phase. Differentiation from asexually replicating stages into non-dividing transmission stages, the gametocytes, takes place inside erythrocytes. Following a period of maturation, micro- and macrogametocytes are ready to initiate transmission when ingested by a mosquito.

The biology of gametocytes shows contrasting features across *Plasmodium* species. *Plasmodium* gametocytes of a majority of species are round and mature in a day or two, whereas gametocytes of *P*. *falciparum*, the species responsible for most malaria-related human deaths, are sickle shaped and reach full morphological maturity in 8–12 days [1]. *P*. *falciparum* gametocytes show a development that is divided into five stages based on morphological changes observed by light microscopy or ultrastructural analyses [2]. In particular, transition to stage II is marked by the formation of a cisternal membrane structure just beneath the plasma membrane called the inner membrane complex (IMC). The IMC acts as a scaffold supporting a dense network of microtubules. By stage IV, the gametocytes are maximally elongated and display pointed ends. Upon transition to stage V, the microtubule network is disassembled and the extremities become more rounded [2].

Terminally differentiated gametocytes resume their development in the mosquito midgut following a blood meal, activated by the presence of xanthurenic acid (XA), a rise in pH, and a drop in temperature [3]. Upon activation both micro- and macrogametocytes increase in size and round up [4] and release the content of secretory vesicles called osmiophilic bodies [5] to possibly aid in the escape of gametocytes from the erythrocyte [6]. Following emergence and activation of translationally repressed mRNAs [7], macrogametes are rapidly available for fertilisation [8]. In contrast, microgametogenesis involves three rounds of genome replication and endomitosis within a single nucleus with the concomitant assembly of eight axonemes. This eventually leads to the release of eight flagellated microgametes in a process called exflagellation [9,10].

Observations by electron and fluorescence microscopy gave general insights into the dynamics of cellular structures during microgametogenesis [4,11–13] (S1 Fig). The mature microgametocyte shows two electron-dense structures that have been linked with the formation of the mitotic spindles and the axonemes, respectively. One of this structure was described as an amorphous MTOC lying on the cytoplasmic face of a nuclear pore that is physically linked to another electron dense aggregation called the intranuclear body in the nuclear face of the same pore [9]. Beside these two terms, different terminologies have been used to describe this gametocyte bipartite MTOC, or parts of it, including spindle pole, spindle pole body, centriolar plaque, or nuclear pole. Upon activation of gametogenesis, the amorphous MTOC gives rise to the basal bodies on which axonemes are nucleated, while the associated intranuclear bodies coordinate the concomitant mitotic events [4]. In this manuscript, we will hereafter use the term basal body to describe the extranuclear part of the gametocyte MTOC nucleating axonemes and intranuclear body for its nuclear counterpart found at each pole of mitotic spindles.

The basal bodies of the forming axonemes remain attached to their respective intranuclear body during the three rounds of mitosis and are moved around the nuclear envelope at each division [14] (S1 Fig). After 1–2 min, the first mitotic spindle is visible and four basal bodies with nucleating axonemes are found in tetrad at each of the two opposing intranuclear bodies. Three to five minutes later, following the assembly of the two spindles of mitosis II, two basal bodies lie at each end of the four intranuclear bodies. By 6–8 min, the four spindles of mitosis III have formed and a single basal body is found attached to each of the eight intranuclear bodies. Chromatin condensation only sets in at the end of mitosis III, when each of the eight short spindles anchors one haploid set of 14 chromosomes to its respective intranuclear body. In *Plasmodium* gametocytes, axoneme polymerisation is intracytoplasmic and independent of intra-flagellar transport [15,16]. Axonemes reach around 14 μm in length in just 10 min and lay coiled around the nucleus. They display a classical organisation with nine doublets of microtubules arranged in a circular pattern surrounding a central pair of singlet microtubules. At the onset of exflagellation, the axonemes become motile and swim basal body first out of the parental cell [4]. The prior attachment of each basal body to an intranuclear body allows each axoneme to drag a haploid genome into the forming flagellated gamete [16].

Available data on the structural organisation and the biogenesis of the *Plasmodium* MTOC coordinating mitosis is very limited. In asexual blood stages, the MTOC is called the centriolar plaque (also named centrosome) that shows a bipartite organisation, with an extranuclear region containing centrin and an intranuclear DNA-free region harbouring microtubule nucleation sites [17]. It appears as an electron-dense area that neither shows a centriole nor any other known distinct structures such as the yeast intranuclear body, the *Dyctiostelium* nucleus associated body or the diatom microtubule centre. In microgametocytes, little is known about the intranuclear body and its molecular link to the basal body while the general organisation of the basal body is slightly less elusive. It is around 0.25 μm long. The central pair of axonemal singlet microtubules does not extend into the basal body but is underlaid by an electron-dense region where γ-tubulin was suggested to reside [16]. Radial spokes have been described between this mass and the peripheral microtubules. Despite coding for four centrins, the *Plasmodium* genomes appears to lack many conserved components of the basal body except SAS6, SAS4/CPAP and CEP135 [18–20]. A *P*. *berghei* SAS6-KO clone displayed reduced basal body numbers, axonemal assembly defects and abnormal nuclear allocation [21]. Kinesin-8B was recently shown to reside in the basal body of microgametocytes and is required for bundling of axonemal microtubules [22,23].

Our understanding of the ultrastructural organisation and cell division events of *Plasmodium* microgametocytes has initially relied on EM. It was more recently complemented by fluorescence live microscopy or super-resolution microscopy using multiple markers to infer the dynamic and the molecular composition of observed structures albeit at a lower resolution [11–13,23,24]. Expansion microscopy (ExM) enables fluorophore labelling of proteins linked to a swellable polymer permitting isotropic physical expansion of the sample, which can be imaged using conventional microscopes below the diffraction limited resolution [25]. Different modifications and variations of expansion microscopy have been implemented [25–27]. Here we used two different variations of ExM to visualise ultrastructural changes during gametocyte development and gametogenesis in both *P*. *falciparum* and *P*. *berghei* [28]. This allowed us to observe details of multiple cellular structures that were previously only accessible by electron or super resolution microscopy. In addition, by localising known markers of the basal body, we could refine the description of the structural and molecular organisation for this organelle and its link with the intranuclear body. Using knockout lines of two known basal body proteins, SAS4 and SAS6, we further showed their different roles in axoneme biogenesis and mitotic transitions during microgametogenesis. Furthermore, we show the differential requirement of two kinases in the homeostasis of the atypical bipartite MTOC coordinating axoneme biogenesis with mitosis.

## Results

### U-ExM of developing *P*. *falciparum* gametocytes highlights the subpellicular microtubule network

*P*. *falciparum* gametocytes show five distinct morphological stages during their development. The fine ultrastructural architecture of each stage has been previously thoroughly investigated by detailed conventional EM [4] or more recently by Serial Block Face Scanning EM [29]. To image the intracellular development of gametocytes, we combined ultrastructure expansion microscopy (U-ExM) with α/β tubulin and N-hydroxysuccinimide (NHS) ester labelling [30,31]. NHS-ester probes react with primary amines and are used for bulk proteome labelling. This analysis was facilitated by using the iGP2 line, an inducible gametocyte-producing line that allows for the routine production of large numbers of viable *P*. *falciparum* gametocytes *in vitro* [32].

**Fig 1:**
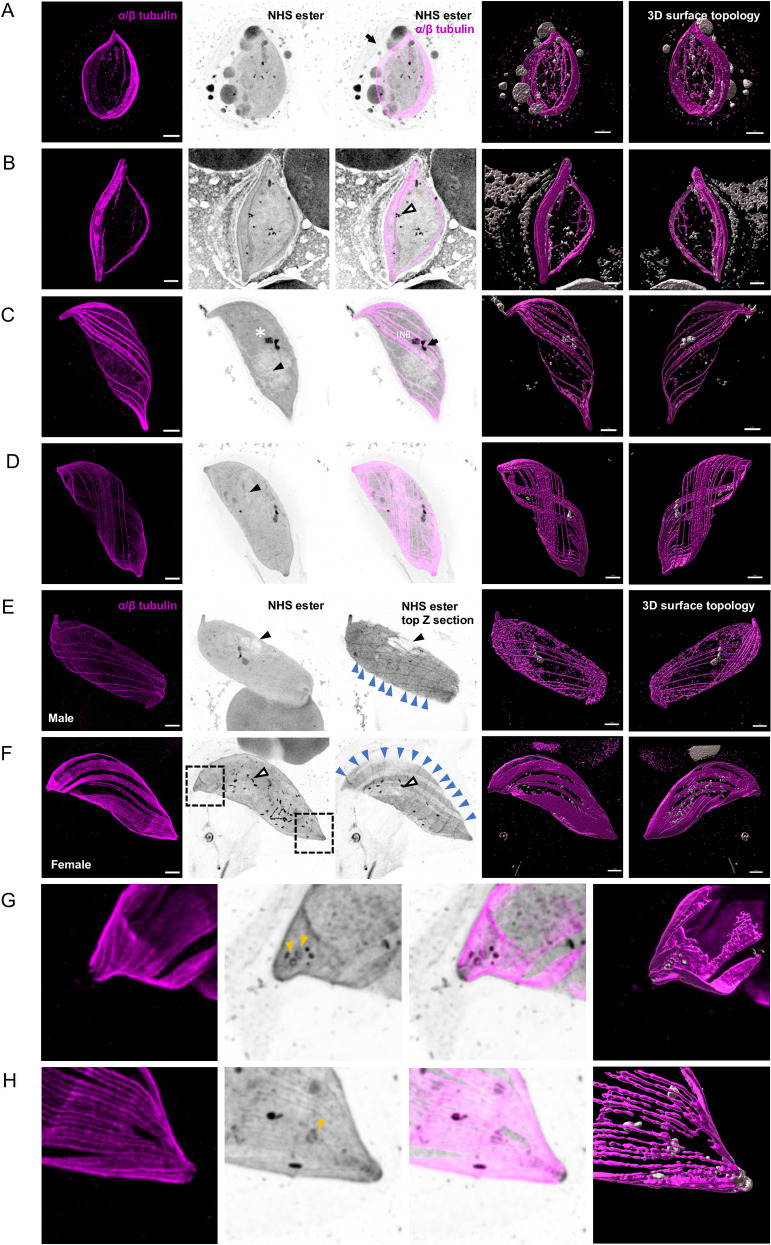
U-ExM allows reconstruction of *P*. *falciparum* gametocytogenesis in 3D. **A-F.** Representative full projections of *P*. *falciparum* gametocyte stages. α/β-tubulin: magenta; amine reactive groups / NHS-ester: shades of grey. Columns 4 and 5 represent the 3D surface topology reconstruction of α/β-tubulin and NHS-ester staining. **A.** A stage III lemon shaped gametocyte. The black arrow shows the host erythrocyte. **B.** A stage III to IV transitioning macrogametocyte, the tips start to get pointed and the cell displays a more elongated shape. Osmiophilic bodies show strong NHS-ester staining as indicated by a white arrow head. **C and D.** A stage IV microgametocyte characterised by the presence of a cytoplasmic amorphous MTOC (black arrow) and the intra nuclear body (INB). The nuclear contour is distinguished by a light NHS-ester staining and the white asterisk indicates the position of the nucleus. Subpellicular microtubules start to surround the gametocyte. **E and F.** Stage V micro- and macrogametocytes displaying rounded ends and an increase in width. Subpellicular microtubules start disassembling but IMC plates remain visible by NHS-ester staining (top Z section, blue arrow heads). In macrogametocytes, NHS-ester dense osmiophilic bodies are visible (white arrow heads). At the gametocyte extremities (dotted square area) 3 to 4 ring-like structures are present. Black arrow heads highlight a region with a lower NHS-ester density that may correspond to the mitochondrion. **G and H**. Close up on the apical ends highlighting NHS-ester dense annuli (orange arrows). Scale bars = 5 μm.

The hallmark of stage II transition is the deposition of the IMC and a dense network of microtubules below the parasite plasma membrane. At this stage, the NHS-ester labelling allowed discrimination of the host erythrocyte from the gametocyte, showing a lighter density in the host cell. In 3 out of 5 infected cells, we observed large NHS-ester dense areas of unknown nature in the host red blood cell. Inside the gametocyte, NHS-ester dense puncta corresponding to osmiophilic bodies were already observed in female gametocytes (Fig 1A). Gametocytes showed a single pointed-end associated with an NHS-ester dense area. This region may correspond to a MTOC as suggested by α/β-tubulin staining that shows a large ribbon of bundled microtubules below the parasite pellicle that loops across the cytoplasm. In stage III gametocytes, the usual lemon shaped organisation was observed, as well as the lateral expansion of the microtubule network. This expansion may coincide with the growth of the previously described 13 Phil1-positive IMC plates [29], however, the plates were not visible by NHS-ester staining at this stage (Fig 1B).

By stage IV, the gametocytes are maximally elongated and display two pointed ends (Fig 1C and 1D). We also noted that the NHS-ester density of the host red blood cell is less marked from this stage. Between the pointed ends, α/β-tubulin staining showed continuous and evenly distributed microtubules running the length of the gametocyte with tightly packed arrays at both extremities. A high NHS-ester density additionally highlighted sutures between the 13 IMC plates suggesting a high protein density between plates (Fig 1E and 1F). Conversely, a low NHS-ester density was observed adjacent to the nucleus (Fig 1E, arrow head). This may correspond to a membrane dense region such as the mitochondrion [2]. Similarly, the nuclear periphery was also highlighted by a lighter NHS-ester staining. The sexual dimorphism became most apparent at this stage: an NHS-ester dense structure spanning the nuclear membrane was observed in all microgametocytes. On the cytoplasmic side, it corresponds to the previously described amorphous electron dense MTOC from which axonemes nucleate (see below) (Fig 1E). On the nuclear side, this NHS-ester dense region likely corresponds to the parental intranuclear body. Upon transition to stage V, the microtubule network progressively disassembled and the tips sequentially became more rounded. As previously described in macrogametocytes, a large number of NHS-ester dense puncta corresponding to osmiophilic bodies were clearly visible (Fig 1F). Intriguingly, NHS-ester dense annuli structures were also observed at both tips of female gametocytes (Fig 1G and 1H).

NHS-ester staining highlighted denser fibres at the extremities of the subpellicular microtubules. We thus wondered whether these structures might correspond to the F-actin cytoskeleton beneath the IMC that was previously described [33]. An antibody against Actin [34] strongly labelled the ends of stage IV macro-and microgametocytes where microtubules did not extend (S2 Fig). Labelling also extended lengthwise into the parasite body alongside the subpellicular microtubules, as previously described [33].

### 3D reconstruction of *P*. *falciparum* and *P*. *berghei* microgametogenesis highlights the spatial dynamics of the microtubule organisation centre coordinating axoneme formation and mitosis

**Fig 2:**
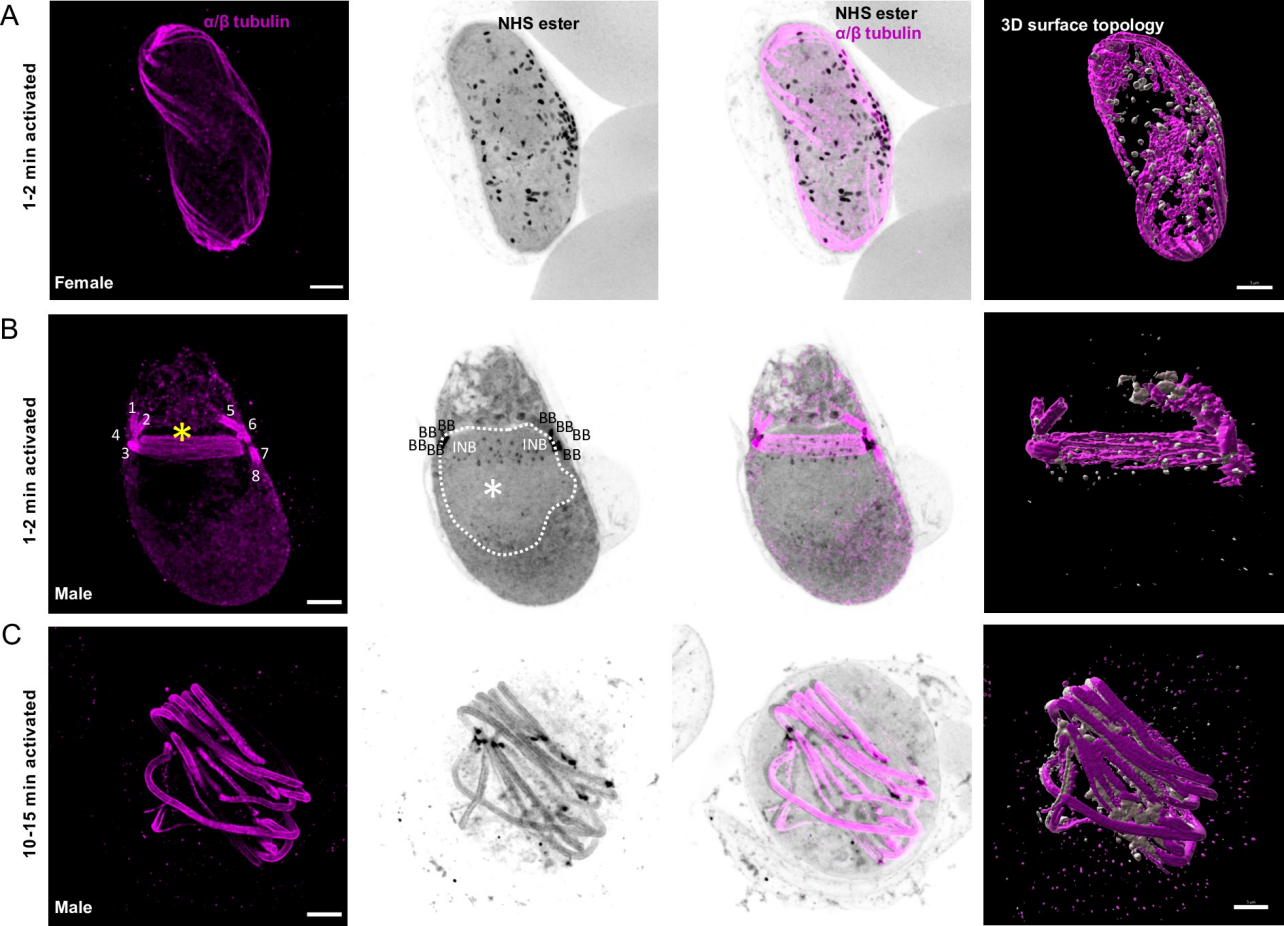
Mitotic structures and axonemes are highlighted by U-ExM during *P*. *falciparum* gametogenesis. **A-C.** Representative full projections of activated *P*. *falciparum* gametocytes. α/β-tubulin: magenta; amine reactive groups / NHS-ester: shades of grey. Column 4 shows the 3D surface topology reconstruction of α/β-tubulin and NHS-ester. **A.** A 1–2 min activated macrogametocyte shows degrading of the subpellicular microtubules and NHS-ester dense osmiophilic bodies. **B.** A 1–2 min activated microgametocyte shows the NHS-ester dense basal bodies (BB) giving rise to axonemes (1 to 8 visible in S1 Movie). The mitotic spindle (yellow asterisk) is highlighted by α/β-tubulin staining and at each extremity the intranuclear bodies (INB) show an NHS-ester dense staining. NHS-ester positive kinetochores are present along the spindle. The white dotted line highlights the nuclear periphery. At this stage, the subpellicular microtubules are completely lost. **C.** A 10–15 min activated microgametocyte displays a round shaped and full length axonemes. Scale bars = 5 μm.

We then investigated the development of *P*. *falciparum* gametocytes into gametes upon activation with xanthurenic acid at a permissive temperature. Two to three minutes post-activation, the remaining subpellicular microtubules were further degraded and the sutures between the IMC plates were not distinguishable anymore (Fig 2A and 2B). At this stage, the surrounding erythrocyte was still visible. In macrogametocytes, osmiophilic bodies moved toward the periphery of the cell. However, no fusion of the vesicles with the parasite plasma membrane were seen by NHS-ester staining (Fig 2A). In microgametocytes, the mitotic spindle was revealed by α/β-tubulin labelling, while the NHS-ester staining showed punctate structures localising on the spindle that likely correspond to kinetochores (Fig 2B) [35,36]. At both extremities of the spindle, two flat intranuclear bodies were strongly labelled by NHS-ester and no overlap with this structure was observed with the α/β-tubulin-positive spindle. On the cytoplasmic face of each intranuclear body, four NHS-ester dense structures that likely include the basal bodies were associated with orthogonally arranged short axonemes (S1 Movie). By ten minutes, microgametocytes have rounded up, undergone three rounds of endomitosis and assembled eight axonemes that are labelled by both NHS-ester and α/β-tubulin (Fig 2C).

**Fig 3:**
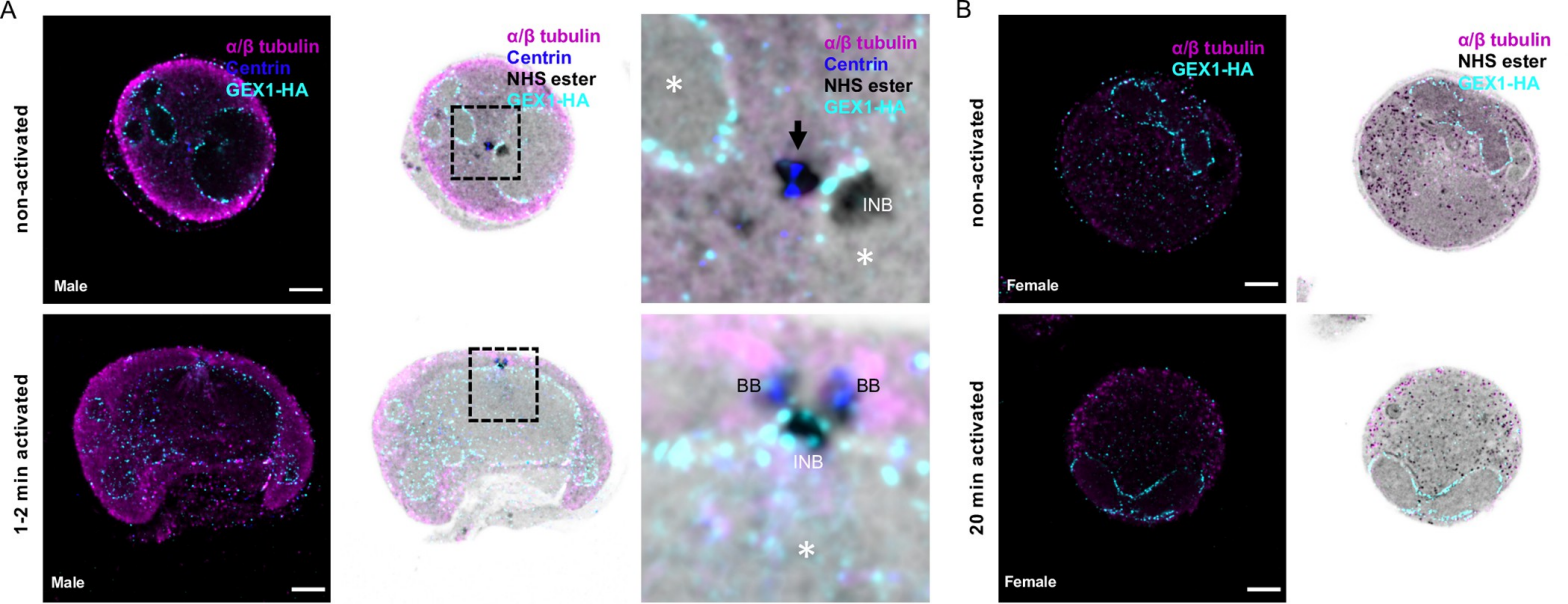
The MTOC coordinating axoneme formation and mitosis shows a bipartite structure across the nuclear membrane during *P*. *berghei* microgametogenesis. **A and B.** Localisation of the nuclear membrane protein GEX1-HA in *P*. *berghei* gametocytes. α/β-tubulin: magenta; amine reactive groups/NHS-ester: shades of grey; centrin: blue; GEX1-HA: cyan. **A.** Non-activated and activated microgametocytes show a lobulated nuclear architecture, as seen by GEX1-HA signal and tubulin negative areas. Column 3 represents a close up of the boxed areas. The nuclear contour is distinguished by a light NHS-ester staining, which matches GEX1-HA staining. Nucleus: white asterisk; amorphous MTOC: black arrow (centrin-positive); basal bodies: BB (centrin-positive); intranuclear body: INB (centrin-negative). **B.** Non-activated and activated macrogametocytes. Macrogametocytes display a crescent-shaped nucleus, very low levels of α/β-tubulin, and NHS-ester dense osmiophilic bodies. Scale bars = 5 μm.

We then applied U-ExM to observe gametogenesis in *P*. *berghei* gametocytes. These have been widely used to investigate the molecular and cellular biology of *Plasmodium* transmission stages. Terminally differentiated *P*. *berghei* microgametocytes were significantly different from their *P*. *falciparum* counterparts, displaying a round shape with no subpellicular microtubules and no IMC plates. While α/β-tubulin labelling did not highlight any particular cellular structure at this stage, NHS-ester staining allowed visualising the host erythrocyte and delineating the contour of the nucleus with a lighter staining. To confirm that the nuclear membrane corresponded to a lighter NHS-ester density, we took advantage of the previously published PbGEX1-HA line [37]. GEX1 was shown to belong to an ancient nuclear envelope protein family, essential for sexual reproduction in eukaryotes. GEX1-HA labelling colocalised with the lighter NHS-ester staining and highlighted a highly lobulated organisation of the nucleus in microgametocytes (Fig 3A). NHS-ester staining also highlighted the osmiophilic bodies in macrogametocytes (Fig 3B) but did not allow the observation of male osmiophilic bodies [38].

**Fig 4:**
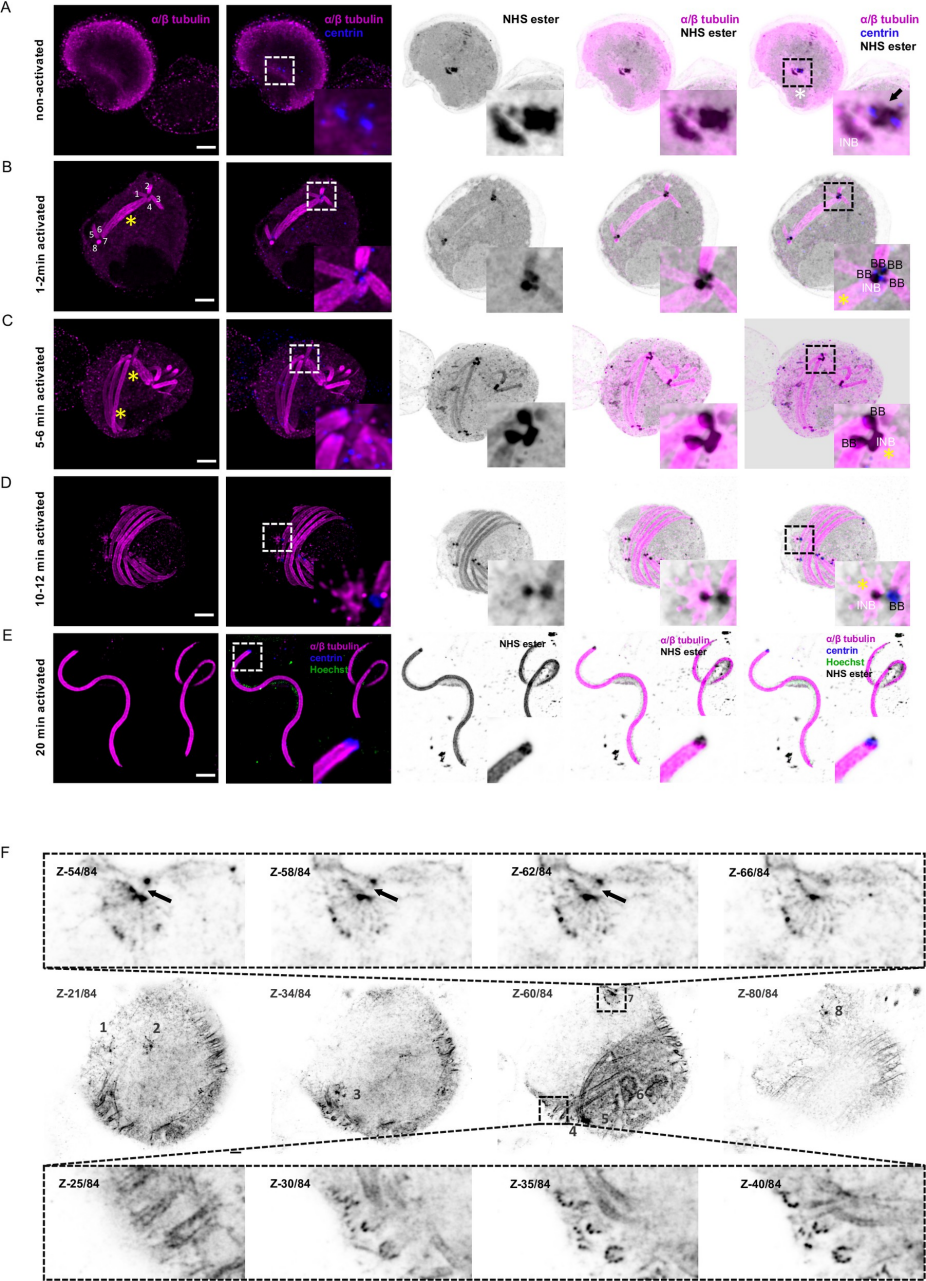
U-ExM and Pan-ExM of *P*. *berghei* microgametogenesis allows further insights into the dynamics of mitotic structures and axoneme formation. **A-E.** Representative full projections of activated *P*. *berghei* gametocytes. α/β-tubulin: magenta; amine reactive groups/NHS-ester: shades of grey; centrin: blue. Boxed areas indicate areas shown in insets. **A.** A non-activated microgametocyte showing an amorphous MTOC (black arrow, centrin-positive) and an intranuclear body (INB, centrin-negative). **B.** A 1–2 min activated microgametocyte, showing two tetrads of basal bodies and eight growing axonemes (1 to 8). In the nucleus the mitotic spindle is highlighted by a yellow asterisk. **C.** A 5–6 min activated microgametocyte is undergoing mitosis II with two mitotic spindles (yellow asterisks). Each intranuclear body (INB) is connected to two cytoplasmic basal bodies (BB). **D.** A 10–12 min activated microgametocyte has undergone mitosis III. Each intranuclear body (INB) is connected to a single cytoplasmic basal body (BB). The yellow asterisk highlights a remnant mitotic spindle. **E.** Exflagellated male gametes display a centrin (blue) and NHS-ester dense basal body at their proximal extremity while DNA (Hoechst: green) runs along the axoneme. **F.** Pan-ExM of a 10–12 min activated and NHS-ester labelled microgametocyte. The central panels represent four different z sections (top left numbers) of the same microgametocyte. Each mitotic spindle is numbered from 1 to 8. Top panels are close ups of four different z sections (top left numbers) of mitotic spindle 7; Arrows indicating the proteinaceous filaments linking the basal body with the intranuclear body. Lower panels show four different z sections (top left numbers) of the boxed area highlighting individual axonemal microtubules. Scale bars = 5 μm.

*P*. *berghei* microgametocytes displayed a bipartite NHS-ester dense structure corresponding to the amorphous MTOC that was partially labelled by an antibody against human Centrin 1. This amorphous MTOC faced the NHS-ester positive intranuclear body that lies on the other side of the nuclear membrane (Fig 4A). Upon activation by xanthurenic acid at a permissive temperature, the cell cycle events of the *P*. *berghei* gametocyte were comparable to *P*. *falciparum* with the sequential segregation and migration of 2x4, 4x2 and 8x1 pairs of intranuclear bodies and centrin-positive basal bodies, from which eight axonemes assemble and grow (Fig 4B–4D). Upon exflagellation, individual male gametes were observed with a strong NHS-ester and centrin-positive basal body at one extremity and a diffuse Hoechst staining of DNA along the axoneme (Fig 4E).

We then asked whether NHS-ester labelling of 2-times expanded cells (Pan-ExM for pan = whole, in Greek), would provide further spatial resolution of the structures highlighted by U-ExM and NHS-ester staining, as previously shown with Hela cells [31]. Sequential expansion allowed to expand *P*. *berghei* gametocytes ~10.0 times. In 15 min activated gametocytes, Pan-ExM allowed resolving individual microtubules of the axonemes and of the mitotic spindle where kinetochores were also NHS-ester positive (Fig 4F). Interestingly, NHS-ester positive filaments linking the intranuclear body and its associated basal body were additionally observed. These proteinaceous filaments may correspond to the structure supporting the cohesion between the intranuclear body and the basal body during microgametogenesis.

### Molecular organisation of the bipartite MTOC made of basal bodies and intranuclear bodies

**Fig 5:**
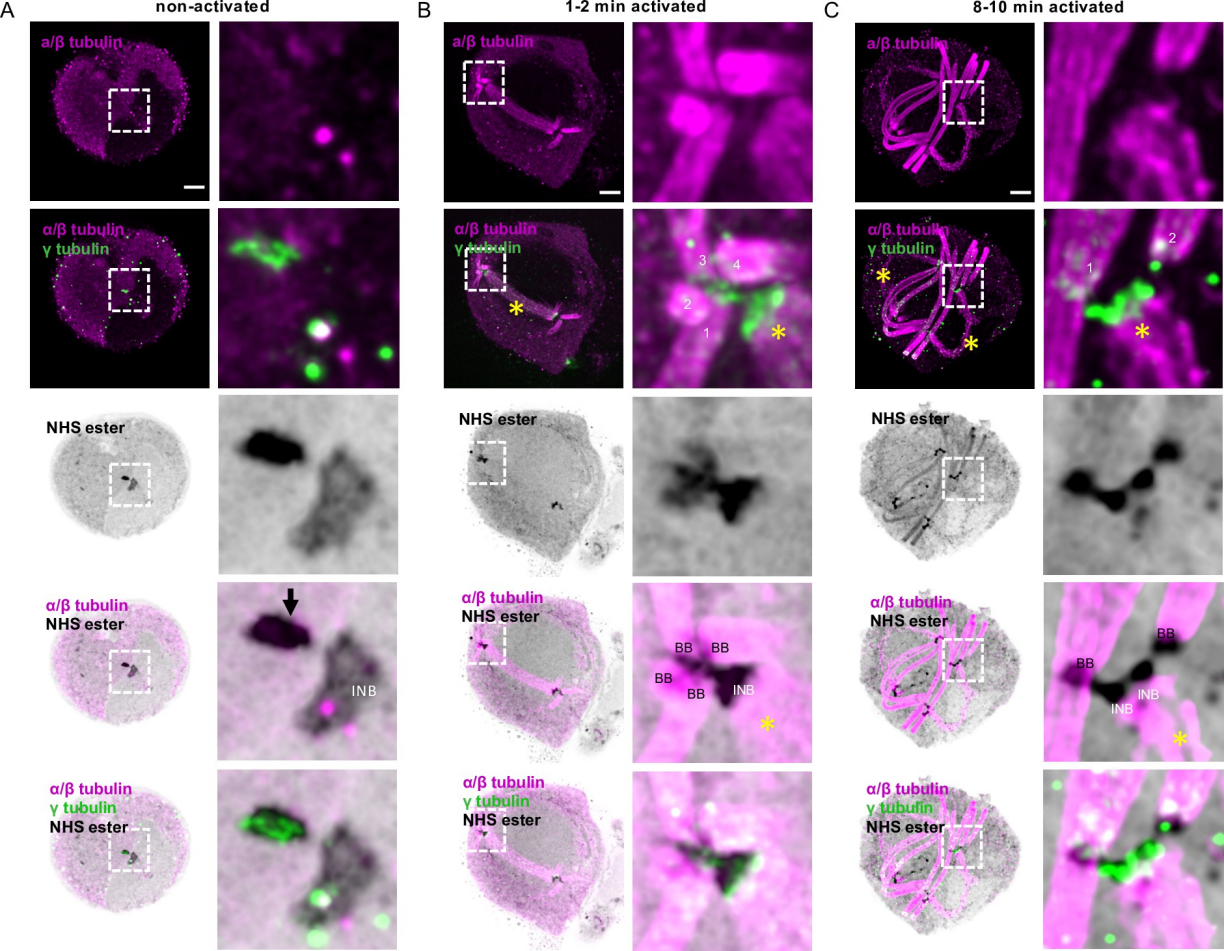
γ-tubulin displays dynamic localisation from the amorphous MTOC to basal bodies and intranuclear bodies during *P*. *berghei* microgametogenesis. **A-C.** Representative full projections of non-activated and activated *P*. *berghei* gametocytes. α/β-tubulin: magenta; amine reactive groups/NHS-ester: shades of grey; γ-tubulin: green. Boxed areas indicate area shown in zoom for each image, highlighting the basal bodies (BB), the amorphous MTOC (black arrow), the intranuclear body (INB) stained with NHS-ester, γ-tubulin and α/β-tubulin. Individual axonemes are numbered 1–4. The yellow asterisks highlight mitotic spindles. Scale bars = 5 μm.

To get further insights into the molecular organisation of the basal bodies and the intranuclear bodies, we set out to map possible markers of these structures on the NHS-ester dense labelling. We first focused on γ-tubulin, which is an indispensable component of MTOCs that contributes to microtubule nucleation and organisation in all eukaryotes (Fig 5) [39]. It is recruited to the centrosome in metazoans or is tethered to the inner and outer plaques of the yeast spindle pole body. Additionally, we 3xHA-tagged two centriolar proteins (S3A–S3D Fig): the proximal protein SAS6 that initiates the cartwheel assembly [40] and SAS4 that surrounds the centriole and facilitates the formation of the microtubule wall [41].

**Fig 6:**
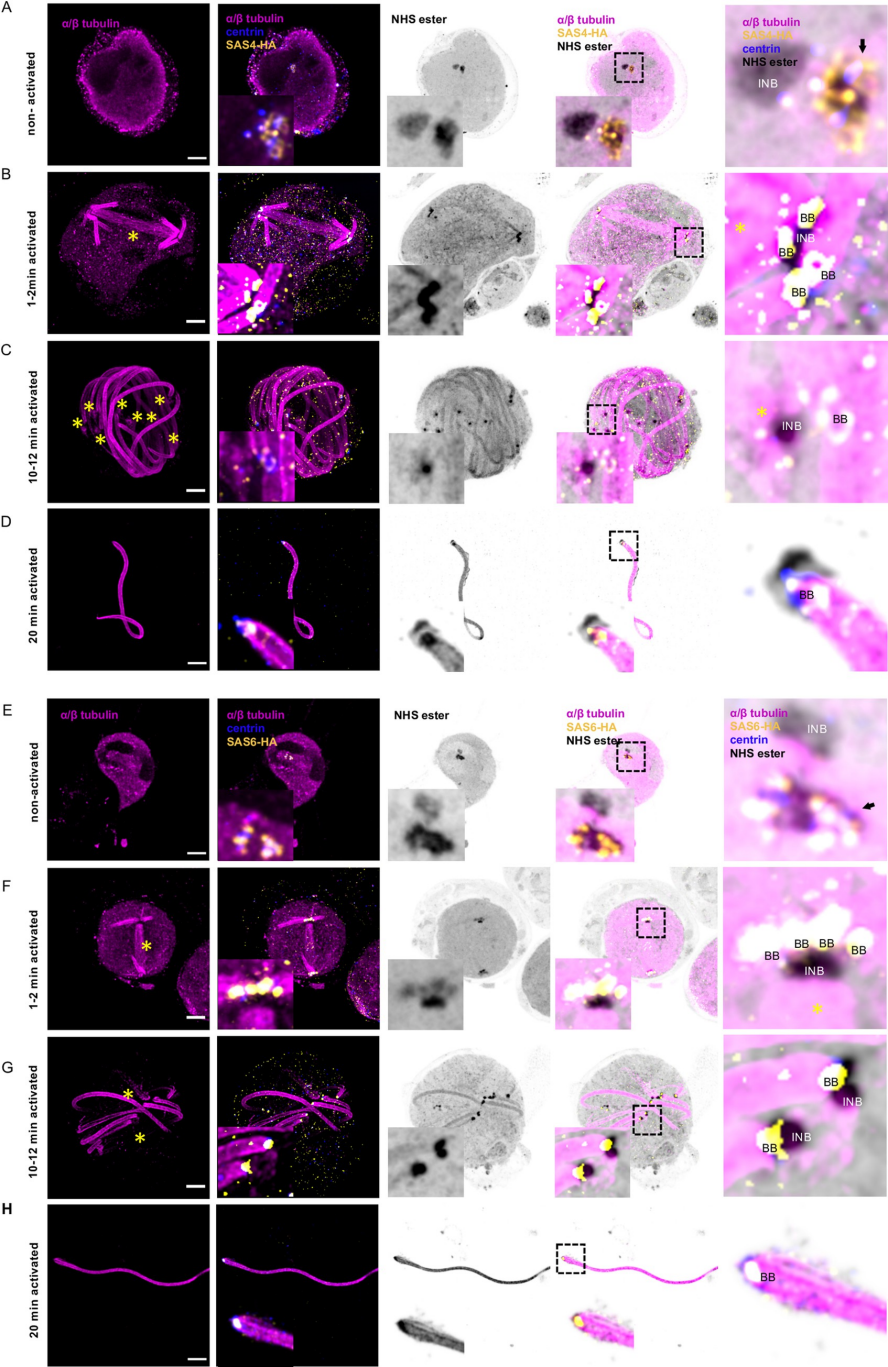
Molecular organisation of the basal body during *P*. *berghei* microgametogenesis. **A-F.** Representative full projections of activated *P*. *berghei* gametocytes. α/β tubulin: magenta; amine reactive groups/NHS-ester: shades of grey; centrin: blue; yellow: SAS4-HA (A-D) or SAS6-HA (E-H). Boxed areas indicate areas shown in insets. Column 5 represents zoomed-in images highlighting the basal bodies (BB), the amorphous MTOC (black arrow), and intranuclear bodies (INB) stained with NHS-ester and for SAS4-HA (A-D) or SAS6-HA (E-H). Scale bars = 5 μm.

**Fig 7:**
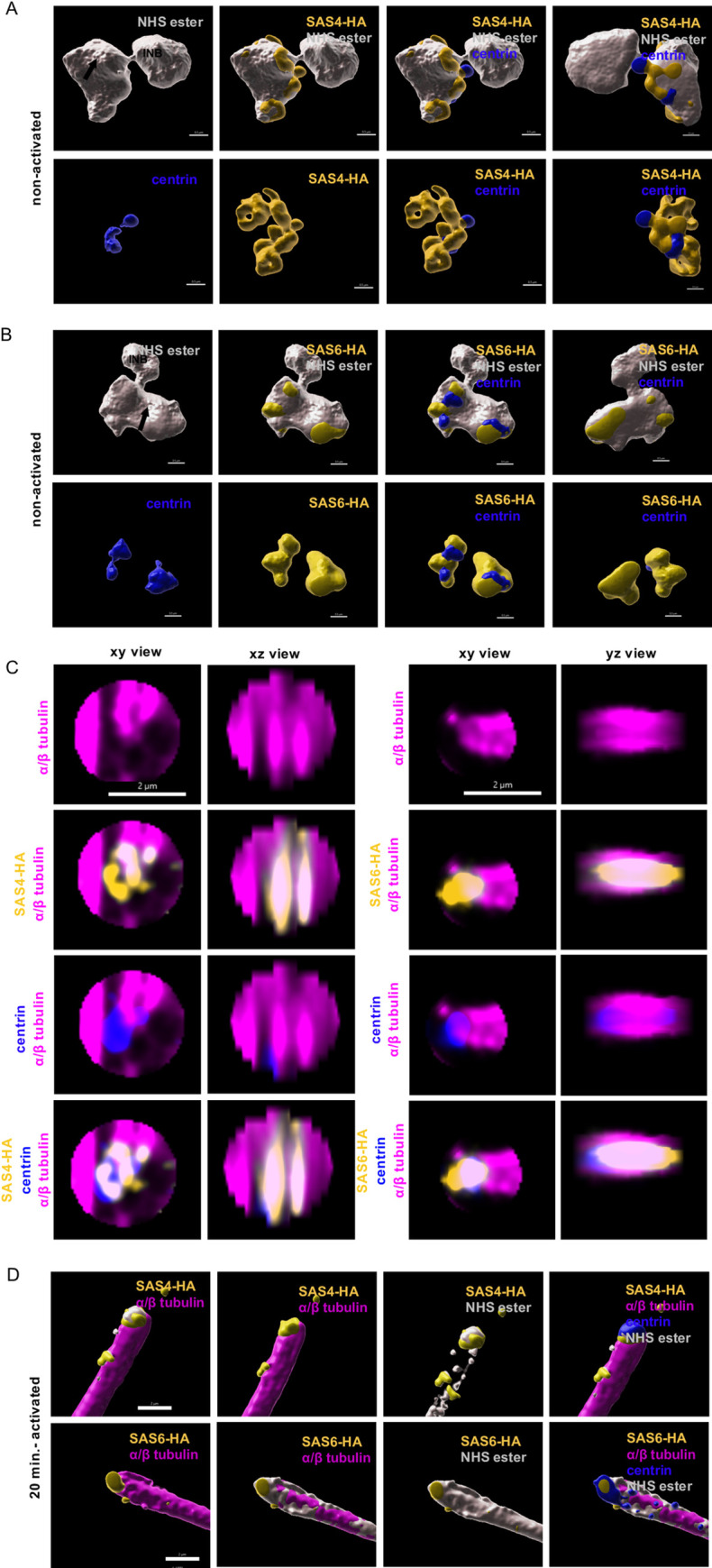
3D reconstruction of the bipartite MTOC during *P*. *berghei* microgametogenesis. **A and B.** 3D surface topology of the amorphous MTOC and intranuclear body of non-activated microgametocytes shown in Fig 6. Amine reactive groups/NHS-ester: shades of grey; centrin: blue; yellow: SAS4-HA (A) or SAS6-HA (B); amorphous MTOC: black arrow; intranuclear body: INB. Scale bar = 0.5 μm. **C.** xy and xz or yz views of individual basal bodies in 10–12 min activated microgametocytes, SAS4-HA (left) and SAS6-HA (right). Scale bar = 2 μm. **D.** 3D surface topology of exflagellated microgametes of Fig 6, SAS4-HA (top row) and SAS6-HA (bottom row). Scale bar = 2 μm.

Prior to the activation of terminally differentiated microgametocytes, γ-tubulin, SAS4-HA and SAS6-HA were only detected in the amorphous MTOC but not in the intranuclear body (Figs 5A, 6A and 6E). At this stage, we could not observe structures resembling basal bodies, as previously described [4]. However, surface topology modelling indicated a certain level of organisation within the amorphous MTOC, where SAS4-HA was distributed at the periphery of the NHS-ester dense structure (Figs 6A and 7A), while SAS6-HA patches were distributed around centrin-positive regions (Figs 6E and 7B). These observations indicate that despite the absence of basal bodies in non-activated gametocytes, their molecular components already display specific localisation patterns within the amorphous MTOC.

In 1–2 min activated gametocytes, a tetrad of four adjacent basal bodies in proximity of the two intranuclear bodies was visible. SAS4-HA and SAS6-HA were localised to NHS-ester dense basal bodies (Fig 6B and 6F). At the proximal end of the axoneme, the diameter of the microtubule wall narrowed down from 220 nm to 120 nm following correction with the 4.2x expansion factor. This section may correspond to the disappearance of the two intra-axonemal microtubule singlets [4]. At the base of the axoneme, a 71 nm long centrin cylinder was observed inside the microtubule wall. A slightly longer SAS6-HA cylinder co-localised with centrin. Around the microtubule wall, SAS4-HA displayed a 105 nm long toroid shape at the base of the centrin/SAS6 cylinder (Fig 6B). At this stage, γ-tubulin was sparsely localised at the extremity of the newly formed basal bodies but was enriched between the NHS-ester dense intranuclear body and the nucleation tip of spindle microtubules (Fig 5B).

In 10–12 min activated gametocytes, a similar organisation was observed with eight pairs of intranuclear bodies and basal bodies observed at the extremity of fully formed axonemes (Fig 6C and 6G). At that stage, a faint γ-tubulin signal could be detected around the basal bodies while a strong signal was observed between the intranuclear body and the mitotic spindles. This suggested a relocalisation from the basal bodies to the intranuclear body during gametogenesis (Fig 5C). In exflagellated microgametes a similar organisation of the basal body was observed with a SAS4-HA toroid surrounding a centrin/SAS6 cylinder (Figs 6D, 6H and 7D).

Of interest, we noted that in the SAS6-HA line, extra SAS6-HA/NHS-ester structures were occasionally observed at the proximal end of single axonemes. A more pronounced phenotype was observed in a SAS6-GFP line, where the NHS-ester dense amorphous MTOC showed an abnormal structure fully labelled with SAS6-GFP. This was associated with aberrant numbers of basal bodies and defective bundling of axonemes (S3E Fig), as previously observed in an independent SAS6-KO line [21]. These observations suggested that the accessibility of the SAS6 C-terminus is important for the correct homeostasis of the basal body (Fig 6G).

### Basal body assembly and axoneme formation show differential requirements for SAS4 and SAS6

**Fig 8:**
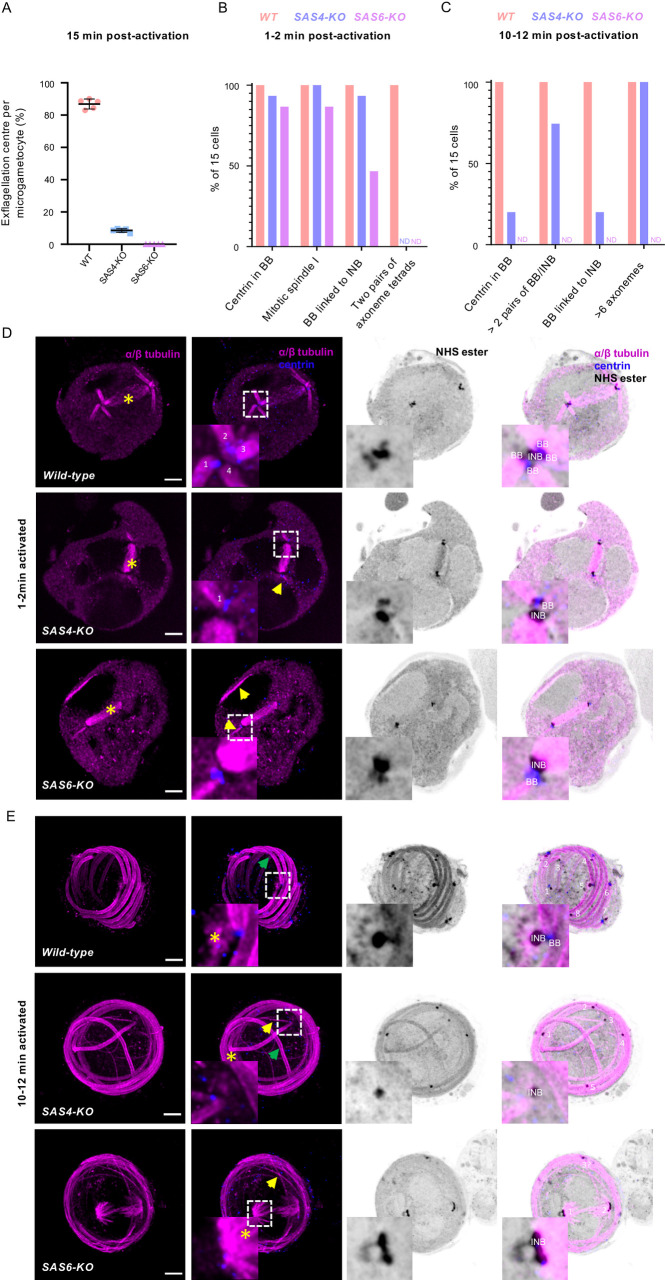
Characterisation of SAS4-KO and SAS6-KO lines highlights different requirements of both proteins for the integrity of the basal body. **A.** Deletions of *sas4* or *sas6* lead to a profound defect in exflagellation (error bars show standard deviation from the mean). **B-C.** Quantification of the phenotypes shown in D and E in 15 individual cells for each line. BB = basal body; INB = intranuclear body. ND = Not Detected. **D-E.** Representative full projections of (D) 1–2 min activated and (E) 10–12 min activated microgametocytes; wild-type (1^st^ row), SAS4-KO (2^nd^ row) and SAS6-KO (3^rd^ row). α/β-tubulin: magenta; amine reactive groups/NHS-ester: shades of grey; centrin: blue. Boxed areas correspond to close-ups. Yellow star = mitotic spindle; yellow arrow = non-bundled microtubules; green arrow = bundled microtubules; INB = intranuclear body; BB = basal body. Scale bar = 5 μm.

We then took advantage of U-ExM to further investigate the requirements for SAS4 and SAS6 during microgametogenesis. To do so, we generated SAS4-KO and SAS6-KO clonal lines (S4A Fig). We first confirmed the importance of SAS6 in microgametogenesis as no exflagellation centres were observed 15 min post-activation in the SAS6-KO line, as previously described [21]. Deletion of *sas4* led to a 90% decrease in the formation of active exflagellation centres (Fig 8A).

We then examined microtubule structures and the MTOC organisation by U-ExM in non-activated and activated microgametocytes (1–2 min, 5–6 min and 10–12 min post-activation) in 15 cells for each line (Figs 8 and S4). No difference could be detected between non-activated SAS4-KO and WT microgametocytes, both presenting an NHS-ester dense intranuclear body and an amorphous MTOC (S4B Fig). The amorphous MTOC stained positive for centrin and γ-tubulin in the KO line, as observed in the WT (S4B Fig). In 1–2 min activated SAS4-KO microgametocytes, we observed an apparently normal spindle of mitosis I. However, only one or two short bundles microtubules were observed at each extremity, as opposed to the two tetrads of axonemes observed in the WT line. At this stage, the bipartite organisation of the MTOC was nevertheless similar between WT and SAS4-KO cells, as revealed by NHS-ester and centrin staining (Fig 8B and 8D). Five to six minutes post-activation, both bundled and non-bundled axonemes were mainly observed but the organisation of the basal body appeared to be compromised with altered NHS-ester and centrin staining (S4C Fig). In 10–12 min activated SAS4-KO microgametocytes, the majority of the axonemes appeared bundled. However, a lower number of NHS-ester and centrin positive basal bodies were observed with only 20% of cells showing an intranuclear body linked to a distinguishable basal body (Fig 8C and 8E). Altogether, this suggests that SAS4 is not essential for axoneme formation, however, its absence leads to defects in the molecular organisation of the basal body during the course of microgametogenesis.

As for the SAS4-KO line, no major differences were observed between non-activated SAS6-KO and WT microgametocytes (S4B Fig). However more dramatic defects were observed upon activation. In 1–2 min activated SAS6-KO microgametocytes, we observed the spindle of mitosis I flanked by apparently normal intranuclear bodies. However, only one or two short non-bundled microtubules originated from only one of the pairs of the corresponding basal bodies. This was associated with reduced numbers of basal bodies at each side and a diffuse centrin staining. Stranded microtubules were also observed in two cells, which were mainly found adjacent to the nuclear membrane (Fig 8B and 8D). In 10–12 min activated microgametocytes, axonemal microtubules increased in length but did not bundle, as previously described [21]. However, we additionally noticed that on the nuclear side, only two intranuclear bodies were facing each other as opposed to the 8 intranuclear bodies in the WT line (Fig 8C and 8E). Interestingly, a large numbers of remnant microtubules of the mitotic spindle were observed suggesting a block prior to entry into mitosis II, a phenotype that was already apparent 5–6 min post-activation (S4C Fig). This was associated with the disappearance of the centrin staining in 90% of observed basal bodies. Altogether this suggests that SAS6 is essential for the *de novo* formation of the eight basal bodies but is also required to maintain the structural integrity of the gametocyte MTOC required for the transition between mitosis I and II.

### U-ExM reveals early roles of SRPK1 and CDPK4 in the homeostasis of the gametocyte MTOC

The visualisation of the MTOC by U-ExM and bulk proteome labelling opened the possibility to reassess the role of previously well characterised kinases required for the formation of microgametes. We focused on CDPK4 and SRPK1 that were reported to cooperate in the early events of microgametogenesis [42].

**Fig 9:**
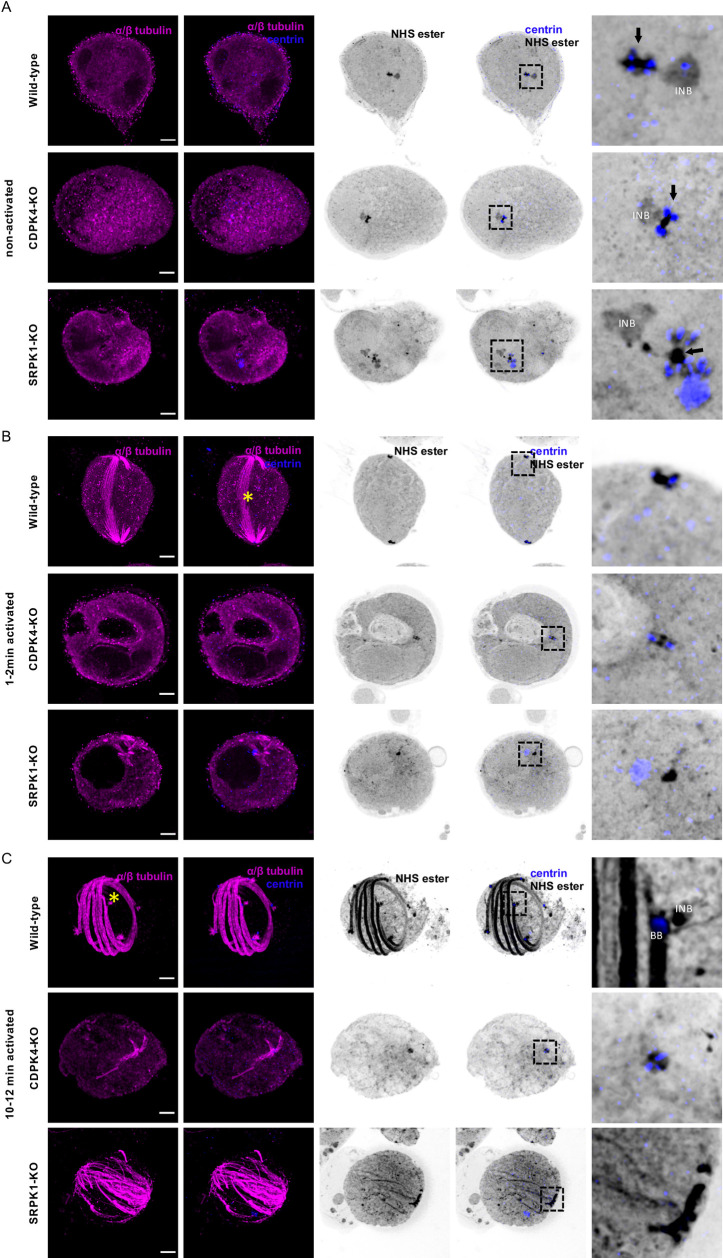
Formation of the amorphous MTOC and its differentiation into functional basal bodies relies on two kinases: CDPK4 and SRPK1. **A-C.** Representative full projections of (A) non-activated, (B) 1–2 min activated and (C) 10–12 min activated microgametocytes; wild-type (1^st^ row), CDPK4-KO (2^nd^ row) and SRPK1-KO (3^rd^ row). α/β-tubulin: magenta; amine reactive groups/NHS-ester: shades of grey; centrin: blue. Boxed areas correspond to close-ups shown in panel 5. Amorphous MTOC: black arrow; intranuclear body: INB; basal body: BB; mitotic spindle: yellow asterisk. Scale bars = 5 μm.

We first focused on CDPK4 that is known to play multiple roles during microgametogenesis including entry into S-phase, formation of mitotic spindles, and assembly of axonemes [43,44]. In non-activated microgametocytes, no obvious difference could be observed between CDPK4-KO and WT cells (Fig 9A). Two minutes upon activation by xanthurenic acid, WT microgametocytes displayed the first mitotic spindle with four growing axonemes at each end. In all five analysed CDPK4-KO microgametocytes, both the parental intranuclear body and the amorphous MTOC appeared unchanged as seen by NHS-ester and centrin labelling. As a consequence, no mitotic spindle nor tetrads of axonemes were observed (Fig 9B). Despite the previously reported defect in DNA replication and the absence of mitotic spindle [43,44], we observed an increase in the nuclear volume as observed in the WT control. However, 10–12 min after activation, 3–4 bundles of axonemes nucleating from a single MTOC were observed in the 5 analysed cells. These observations indicated that despite the defective reorganisation of the amorphous MTOC, microtubule nucleation was induced but defective in the absence of CDPK4 (Fig 9C).

In the SRPK1-KO line, more obvious ultrastructural defects were observed prior to activation (Fig 9A). In non-activated SRPK1-KO microgametocytes, the intranuclear body was absent in 4 out of 5 cells and was detached from the amorphous MTOC in the remaining cell. Additionally, centrin was found in 6–8 NHS-dense spheroids surrounding the amorphous MTOC with one larger centrin positive spheroid in all analysed cells. Upon activation, we did not observe segregation of the basal bodies and the intranuclear bodies (5/5 cells). Despite these early structural defects, two tetrads of nucleating axonemes remained in a close position and no mitotic spindle could be observed between these two tetrads (Fig 9B). Although a faint centrin signal was visible at the proximal end of each basal body, centrin mainly remained in the single large spheroid next to the eight basal bodies (Fig 9B). Axonemal microtubules did nucleate, but they did not seem to bundle and remained disorganised in the cytoplasm. In 10–15 min activated microgametocytes, similar defects were observed, with most basal bodies remaining closely clustered. Axonemal microtubules further elongated but lacked a clear axonemal organisation, as observed in the parental line (Fig 9C). Altogether, these results reveal a role for SRPK1 in the homeostasis of the MTOC in *Plasmodium* microgametocytes.

## Discussion

We have recently implemented U-ExM to visualise cytoskeletal structures in various stages of *Plasmodium* parasites [28]. This revealed that a divergent and reduced form of a conoid is present. This organelle is required for host cell invasion by apicomplexan parasites, and was thought to be lost in *Plasmodium*. Here we first combined U-ExM with bulk proteome labelling to investigate cellular structures in *Plasmodium* gametocytes, the parasite forms that initiate the parasite transmission to the mosquito. NHS-ester staining allowed to resolve numerous structures that were only accessible by EM or super-resolution microscopy such as the sutures between the IMC plates, the osmiophilic bodies, the contour of the nucleus or the host erythrocyte. We additionally observed NHS-ester dense annuli at the extremities of *P*. *falciparum* gametocytes that are reminiscent of the apical annuli recently described in *Toxoplasma gondii* tachyzoites [45]. Of particular interest, bulk proteome labelling allowed visualising numerous structures involved in the cell cycle, including the amorphous MTOC and the intranuclear body in non-activated microgametocytes. Upon activation bulk proteome labelling further highlighted the dynamics of the basal and intranuclear bodies, cytoplasmic axonemes, mitotic spindles and kinetochores. Pan-ExM improved the resolution of these structures, however, unlike U-ExM, we were unable to label individual proteins, as was done in other cell types [30,31]. In addition, we found Pan-ExM to be experimentally a lot more demanding than U-ExM. Altogether these observations indicate that the combination of U-ExM with bulk proteome labelling represents an accessible approach to study the ultrastructural context of *Plasmodium* cells with a conventional confocal microscope and will nicely complement electron microscopy or live-cell imaging studies [46,47].

**Fig 10:**
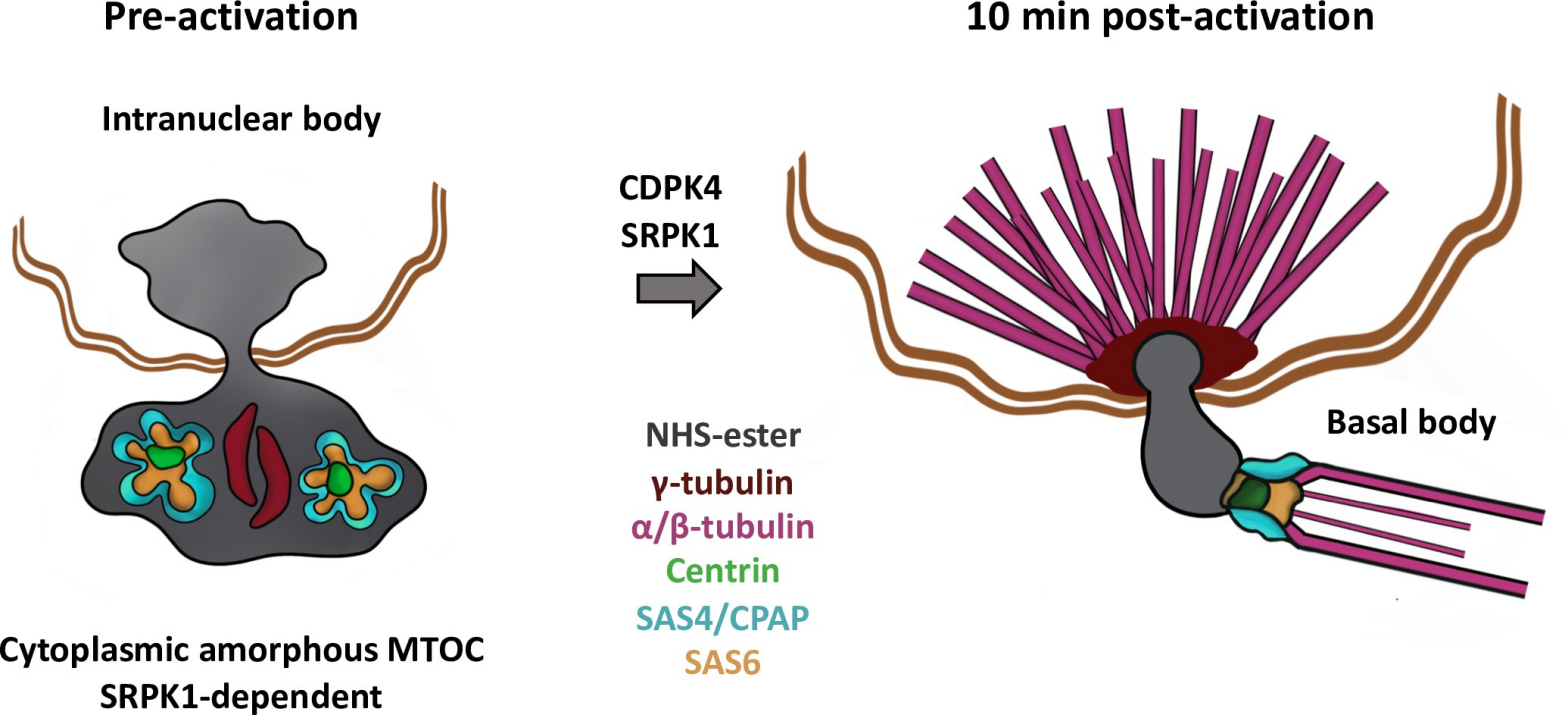
Working model showing the molecular organisation and dynamics of the bipartite *Plasmodium* MTOC coordinating mitosis and axoneme formation during microgametogenesis. In the non-activated microgametocyte the intranuclear body is connected to the amorphous MTOC across the nuclear membrane. The amorphous MTOC corresponds to a deuterosome-like structure where components of the basal body and associated proteins localise in distinct or overlapping subdomains. The molecular organisation of the amorphous MTOC and its link with the intranuclear body relies on SRPK1. Upon activation of CDPK4, the intranuclear body gives rise to eight intranuclear bodies that coordinate the assembly of mitotic spindle while the amorphous MTOC differentiates into eight basal bodies to form eight axonemes (one representation shown). γ-tubulin shows dynamic localisation from the amorphous MTOC to the basal bodies and the intranuclear bodies to sequentially nucleate microtubules of the axonemes and mitotic spindles.

We then exploited the possibility to combine U-ExM with classical immunostaining to clarify the elusive nature of the MTOC in *Plasmodium* microgametocytes (Fig 10). In non-activated gametocytes the NHS-ester dense intranuclear body and amorphous MTOC were connected through the nuclear membrane by proteinaceous filaments. At that stage, the two structures likely host the molecular components required for the formation of the intranuclear bodies and the basal bodies required during microgametogenesis. While none of the proteins investigated could be mapped into the intranuclear body in non-activated microgametocytes, we show that centrin, γ-tubulin, SAS4-HA and SAS6-HA are present in the amorphous MTOC. At this stage, the localisation of centrin, SAS4-HA and SAS6-HA did not reveal structures resembling basal bodies, as previously suggested by EM [4]. However, the distribution of each protein suggested the presence of defined subdomains within the amorphous MTOC showing two elongated γ-tubulin subdomains, two to four centrin regions surrounded by patches of SAS6 and SAS4. This organisation is reminiscent of deuterosomes that serve as platforms to produce basal bodies in a mother centriole-independent manner in multiciliated cells. Deuterosomes are electron dense structures displaying nested subdomains of proteins, including γ-tubulin and SAS6 [48,49]. Upon activation of microgametocytes, we confirmed the rapid *de novo* assembly of two tetrads of four basal bodies from the amorphous MTOC through the dynamic localisation of SAS4, SAS6, centrin and γ-tubulin. Upon activation, γ-tubulin relocalises rapidly at the interface between the mitotic spindle and the intranuclear body, in a pattern that is reminiscent of the multi-layered structures of the yeast spindle bodies [50]. Identification of components of the intranuclear bodies will likely shed light on the molecular organisation of the intranuclear body and its replication.

Combining U-ExM with localisation of known basal body markers allowed us to refine the structural organisation of this organelle in *P*. *berghei*. Interestingly, NHS-ester and centrin staining suggests a similar organisation between *P*. *berghei* and *P*. *falciparum* although some differences may be highlighted by future analyses. Upon activation, centrin and SAS6 localised in the proximal lumen of the newly formed microtubule wall, while SAS4 formed a toroid surrounding this structure. In the human centriole, SAS6 is important for the cartwheel formation that lies below Centrin-2. In *Plasmodium*, SAS6 and centrin largely co-localised suggesting that the proximal region and the central core are compacted in a single structure where centrin may not only serve as a scaffolding protein but as a platform for the axoneme nucleation. In the absence of SAS6, the basal body formation is strongly impaired and centrin is not maintained at the basis of the nucleating microtubules. Surprisingly, SAS4 was also found to be important to maintain the basal body structure later during microgametogenesis but was not required to form apparently bundled axonemes. This suggests that SAS4 is not essential in the assembly of a functional basal body but is required later to maintain its structural organisation. Surprisingly while SAS4 and SAS6 are not required for mitosis in asexual blood stages, deletion of *sas6* showed a block between mitosis I and II. It remains unknown whether this is due to a direct role of both proteins in the replication of the intranuclear body or whether the loss of the basal bodies on the cytoplasmic side of the MTOC indirectly affects progression to mitosis II. We also confirmed that C-terminal tagging of SAS6 affects the homeostasis of the basal body suggesting the importance of protein-protein interactions in the assembly of this structure.

The known proteome of the *Plasmodium* basal body [16] is currently limited compared with other eukaryotes where up to 100 proteins have been assigned to the centriole [51,52]. So far, only centrins, SAS6, SAS4 and CEP135 [18–20] have been detected in the *Plasmodium* genome. Interestingly, the latter three proteins are part of the minimal set of proteins required for the nine-fold symmetry of basal bodies [53]. Further biochemical and functional studies will be required to determine whether additional proteins are involved in the assembly and function of the *Plasmodium* basal body.

The structural organisation of the centriolar plaque has recently been refined in asexually replicating blood stages [17,54,55] that undergo endomitosis but without axoneme nucleation. In these stages, the centriolar plaque also shows a bipartite organisation with an extranuclear region containing centrin that is linked through a nuclear pore to an intranuclear chromatin-free region harbouring microtubule nucleation sites. In the microgametocyte, a similar organisation is observed, however, centrin is incorporated into basal bodies and likely serves as a link to coordinate mitosis with axoneme formation. The shuttling of γ-tubulin from the basal bodies to the intranuclear bodies likely reflects the sequential nucleation of axonemes and mitotic spindles from this unique bipartite MTOC. In eukaryotes, centrioles are highly conserved and fulfil important cellular functions such as the nucleation of axoneme as a basal body or the organisation of pericentriolar material to form the centrosome. Phylogenetic analyses suggested that the basal body function of the centriole is ancestral whereas its requirement in the organisation of the pericentriolar material emerged later [53,56]. The coupling of mitosis with axoneme formation in *Plasmodium* microgametocytes may reflect a transitory state in the evolution of a basal body function. It also shows functional similarities with the basal body-dependent segregation of the mitochondrial or kinetoplast DNA during the cell cycle of *Trypanosoma brucei* [57]. In the absence of axoneme requirement in the *Plasmodium* asexual blood stages, it is possible that the expression of basal body components is repressed or was lost in these stages but that the centriolar plaque retained the bipartite structural scaffold to fulfil mitosis.

Kinase activity has long been known to be crucial for the regulation of basal body homeostasis. A central kinase for basal body biogenesis is the polo-like kinase 4 (PLK4) in humans or related orthologous kinases in other eukaryotes [58]. PLKs were proposed to be present in the cenancestor of eukaryotes but lost in some lineages [56] including *Plasmodium* [59,60]. It was suggested that in these lineages, either the basal body duplication does not require kinase activity or that other kinases fulfil that role. Here we analysed with U-ExM two previously characterised kinase mutants, SRPK1 and CDPK4, in which mitotic defects during microgametogenesis were observed [42,44,59]. A SRPK1-KO line produced morphologically normal microgametocytes despite substantial deregulation of phospho-dependent pathways prior to activation [28]. Here U-ExM reveals structural defects of the amorphous MTOC in terminally differentiated SRPK1-KO gametocytes, confirming the role of SRPK1 prior to activation. We additionally show that deletion of *srpk1* is linked with misincorporation of centrin in the amorphous MTOC leading to defects in basal body segregation and axoneme bundling. While U-ExM did not allow us to define precisely subdomains in the amorphous MTOC of WT parasites, the mislocalisation of centrin in the SRPK1-KO line further supports the presence of a defined molecular architecture within the amorphous MTOC. Deletion of *cdpk4* was previously shown to lead to a strong reduction in the formation of mitotic spindle and axonemes [14,43]. We further show that both defects are linked to an early block in the *de novo* formation of the eight basal bodies from the amorphous MTOC. Despite this marked defect, the formation of short microtubules is observed at later time-points suggesting that other regulatory mechanisms are at play to induce microtubule formation upon activation of microgametocytes. Our previous phosphoproteome analyses of SRPK1-KO and CDPK4-KO in gametocytes suggested that these kinases regulate similar biological processes linked to the microtubule skeleton in gametocytes [42]. Notably, the specific phosphosites that were disrupted did not overlap between mutants, suggesting that the two protein kinases have different sets of substrates and possibly regulate different aspects of the MTOC homeostasis, possibly explaining the different phenotypes observed between the two mutants. However, in activated gametocytes, we previously found that SRPK1 is phosphorylated in a CDPK4-dependent manner suggesting a direct interplay between the two kinases [42]. Additional layers of regulation mediated by ubiquitination may also be involved in the homeostasis, as previously suggested [61,62], and U-ExM will likely represent an instrumental approach to further address this field of research.

In this work, the high resolution of U-ExM combined with the ease of labelling individual proteins has allowed us to gain new insights into the dynamic molecular organisation of an atypical bipartite MTOC coordinating mitosis and axoneme formation in a non-model eukaryote. Deeper characterisation of its molecular composition and its homeostasis may in the future help highlighting general principles underlying the evolution and organisation of MTOCs in eukaryotes.

## Materials and methods

### Ethics statement

All animal experiments were conducted with the authorisation number GE/58/19, according to the guidelines and regulations issued by the Swiss Federal Veterinary Office.

### *P*. *berghei* maintenance and gametocyte production

*P*. *berghei* ANKA strain-derived clone 2.34 [43] together with derived transgenic lines GEX1-HA [63], SAS4-HA, SAS6-HA, CDPK4-KO [43] and SRPK1-KO [59] were grown and maintained in CD1 outbred mice as previously described [64]. Six- to ten-week-old mice were obtained from Charles River Laboratories (France), and females were used for all experiments. Mice were specific pathogen free (including *Mycoplasma pulmonis*) and subjected to regular pathogen monitoring by sentinel screening. They were housed in individually ventilated cages furnished with a cardboard mouse house and Nestlet, maintained at 21 ± 2°C under a 12-hour light/dark cycle, and given commercially prepared autoclaved dry rodent diet and water ad libitum. The parasitaemia of infected animals was determined by microscopy of methanol-fixed and Giemsa-stained thin blood smears.

Gametocyte production and purification was performed as previously described [64]. Parasites were grown in mice that had been phenyl hydrazine-treated 3 days before infection. One day after infection, sulfadiazine (20 mg/L) was added in the drinking water to eliminate asexually replicating parasites. For gametocyte purification, parasites were harvested in suspended animation medium (SA; RPMI 1640 containing 25 mM HEPES, 5% fetal calf serum [FCS], 4 mM sodium bicarbonate, pH 7.20) and separated from uninfected erythrocytes on a Histodenz cushion made from 48% of a Histodenz stock (27.6% [w/v] Histodenz [Sigma/Alere Technologies, Germany] in 5.0 mM TrisHCl, 3.0 mM KCl, 0.3 mM EDTA, pH 7.20) and 52% SA, final pH 7.2. Gametocytes were harvested from the interface. Gametocytes were activated in RPMI 1640 containing 25 mM HEPES, 4 mM sodium bicarbonate, 5% FCS, 100 μM xanthurenic acid, pH 7.4).

### Generation of targeting constructs and parasite transfection

In order to generate the transgenic SAS4-HA and SAS6-HA lines, library clones PbG02_A-10a01 and PbG03-39b05 from the PlasmoGEM repository (http://plasmogem.sanger.ac.uk/) were used to generate HA tagging vectors, respectively. To generate the transgenic SAS4-KO and SAS6-KO lines transfection vectors PbGEM-338003 and PbGEM-327715 were used. Sequential recombineering and gateway steps were performed as previously described [65,66]. Insertion of the GW cassette following gateway reaction was confirmed using primer pairs GW1 x QCR1 and GW2 x QCR2. Oligonucleotides used in this study are listed in S1 Table. The modified library inserts were then released from the plasmid backbone using NotI. Both the KO and HA targeting vectors were transfected into the 2.34 parasite line, KO lines were first cloned for further experiments. Schizonts for transfection were obtained and parasite transfection was performed as previously described [64].

### *P*. *falciparum* culture

*In vitro* cultures to produce *P*. *falciparum* gametocytes using the iGP2 line was performed as described in [32]. Briefly, the parasite line was grown in human erythrocytes in RPMI-1640 medium with glutamine (Gibco), 0.2% sodium bicarbonate, 25 mM HEPES, 0.2% glucose, 5% human serum, and 0.1% Albumax II (Life Technologies). The medium was complemented with 2.5 mM D-Glucosamine (D-GlcN) to restrict the overexpression of the sexual commitment factor GDV1 under the control of a *glmS* riboswitch. At ring stage parasites, D-GLcN was removed to trigger the expression of GDV1 to initiate gametocytogenesis. Gametocyte maturation was allowed for 10 to 15 days. For the first 6 days following induction, 50 mM GlcNAc was added to the culture medium to eliminate asexual parasites. Parasite development was monitored daily by Giemsa staining.

### U-ExM

Synchronized stage specific *P*. *falciparum* iGP line cultures were pelleted and fixed in 4% formaldehyde for 20 minutes. Sample preparation of *P*. *berghei* parasites for U-ExM was performed as previously described [28], except that 4% formaldehyde (FA) was used as fixative. Key reagents used for ExM are listed in S2 Table. Fixed samples were then attached on a 12 mm round Poly-D-Lysine (A3890401, Gibco) coated coverslips for 10 minutes. Thereafter the following steps were performed: 1. To add anchors to proteins, coverslips were incubated for 5 hours in 1.4% formaldehyde (FA)/ 2% acrylamide (AA) at 37°C, 2. Gelation was performed in ammonium persulfate (APS)/Temed (10% each)/Monomer solution (23% Sodium Acrylate; 10% AA; 0,1% BIS-AA in PBS) for 1 hour at 37°C. 3. Denaturation was performed for 1 hour and 30 minutes at 95°C. 4. Gel expansion and antibody labelling. After denaturation, gels were incubated in ddH2O at room temperature for 30 minutes. Next, gels were incubated in ddH2O overnight for complete expansion. The following day, gels were washed in PBS twice for 15 minutes to remove excess of water. Gels were then incubated with primary antibodies at 37°C for 3 hours, and washed 3 times for 10 minutes in PBS-Tween 0.1%. Incubation with the secondary antibody was performed for 3 hours at 37°C followed by 3 washes of 10 minutes in PBS-Tween 0.1% (all antibody incubation steps were performed with 120–160 rpm shaking at 37°C). Directly after antibody staining, gels were incubated in 1 ml of 594 NHS-ester (Merck: 08741) diluted at 10 μg/mL in PBS for 1 hour and 30 minutes at room temperature on a shaker. The gels where then washed 3 times for 15 minutes with PBS-Tween 0.1% and expanded overnight in ultrapure water. Overnight, a second round of expansion was done in water before imaging. 5. Sample mounting and imaging: 1cm x 1cm gel pieces were cut from the expanded gel and attached on 24 mm round Poly-D-Lysine (A3890401, Gibco) coated coverslips to prevent gel from sliding and to avoid drifting while imaging. The coverslip was then mounted on a metallic O-ring 35mm imaging chamber (Okolab, RA-35-18 2000–06) and imaged.

### Pan-ExM

The pan-ExM procedure was performed as previously published [31]. Briefly, samples were fixed in 4% formaldehyde and attached on a 12 mm round Poly-D-Lysine (A3890401, Gibco) coated coverslips for 10 minutes. Thereafter the following steps were performed. 1. Addition of anchors to proteins: coverslips were incubated for 5 hours in anchoring solution, 1.4% FA/ 2% AA mix at 37°C 2. 1^st^ Gelation: gelation was performed in a gelation chamber, using APS/Temed/ (19% Sodium Acrylate; 10% AA; 0.1% N,N-(1,2-dihydroxyethylene) bis-acrylamide -DHEBA- in PBS) for 1 hour at 37°C. 3. Denaturation: the gel was carefully removed from the gelation chamber and placed in the denaturation buffer for 1 hour and 30 minutes at 76°C. 4. First expansion: after denaturation, a piece of the 1 cm x 1 cm gel was cut and placed in ultra-pure water for about 2 hours; water was changed 2 times for the first 30 min. This leads to an expansion factor ranging from 4x to 4.5x. 5. Re-embedding in neutral hydrogel/2^nd^ gel: 2 cm x 2 cm gels were cut and used for further downstream processes as bigger gels are difficult to handle and require a larger volume of reagents. Water was replaced by the second gelling solution (APS/Temed/10% AA, 0.05% DHEBA) and incubated for 3 x 20 min at room temperature on a shaker. Thereafter the gel was placed in a humid gelation chamber and incubated at 37°C for 2 hours. 5. Addition of anchors to proteins: the gel was removed from the gelation chamber and placed in anchoring solution (1.4% FA/ 2% AA) at 37°C for 5 hours. 6. 3^rd^ gelation: The gel was washed three times in PBS and incubated three times with the third gelation solution APS/Temed/ (19% Sodium Acrylate; 10% AA; BIS-AA in PBS) on ice on a shaker. After the third incubation, the gel was placed on a glass slide and covered on top with a coverslip (22 mm x 22 mm), placed in a humidified chamber and incubated at 37°C for 2 hours. 7. Dissolution of 1^st^ and 2^nd^ gel: the gel was removed from the humidified chamber and incubated with 0.2 M NaOH solution in a 6-well plate for 1 hour at room temperature on a shaker. After 1 hour, the solution was removed, rinsed with PBS and transferred to a beaker with PBS and washed 3 to 4 times for 30 min each or until the pH changed to 7.4. 8. NHS-ester staining: the gel was placed in a 6 well plate and incubated in 594 NHS-ester (Merck, 08741) diluted at 10 μg/mL in 3 ml PBS for 1 hour and 30 minutes at room temperature on a rocking platform. The gels were then washed three times for 15 minutes with PBS-Tween 0.1%. 9. 2^nd^ expansion: gels were placed in a beaker filled with ultra-pure water and incubated for 4–6 hours; water was changed every 30 minutes for the first hour of incubation. 10. Sample mounting and imaging: this step was performed exactly as explained above for U-ExM.

### Image acquisition, analysis and processing

Images were acquired on Leica TCS SP8 microscope with HC PL Apo 100x/ 1.40 oil immersion objective in lightning mode to generate deconvolved images. System optimized Z stacks were captured between frames using HyD as detector. Images were processed with ImageJ, LAS X and Imaris 9.6 software. Imaris software was used for 3D surface reconstruction and xy, xz and yz representations.

## Supporting information

S1 Fig A single MTOC coordinates mitosis and axoneme assembly during *Plasmodium* microgametogenesis. Circulating microgametocytes are arrested at a G0-like stage at the haploid level showing an amorphous MTOC (black arrow) lying on the cytoplasmic face of a nuclear pore that is physically linked to another electron dense aggregation called the intranuclear body (INB) in the nuclear face of the same pore. The molecular organisation of both structures and their link is unknown. One minute after activation, the first genome replication is completed and the spindle of mitosis I (spindle: yellow, kinetochores: pink) is observed between the two intranuclear bodies (INB). At the same time, the amorphous MTOC gives rise to 8 basal bodies (BB) which initiate nucleation of eight axonemes (magenta). By 8 minutes three successive rounds of genome replication and endomitosis have happened and at 10 minutes, full length axonemes become motile. As each basal body remains attached its corresponding intranuclear body, they drag a haploid genome that is incorporated into the exflagellating gametes.
(TIF)

S2 Fig U-ExM confirms the organisation of the actin cytoskeleton during *P*. *falciparum* gametocytogenesis. **A-D**. Representative full projections of *P*. *falciparum* gametocyte stages. α/β tubulin: magenta; amine reactive groups / NHS-ester: shades of grey; actin: cyan. Column 6 shows the 3D surface topology reconstruction of α/β-tubulin and actin. **A and B.** At early stages, a punctate distribution of actin is observed. **C-D.** From stage IV gametocytes, actin shows a polarised localisation, mostly concentrated at both extremities in tubulin-free areas showing a mesh-like organization. Actin also extends lengthwise alongside the subpellicular microtubules. Insets in (C and D) are close ups of both extremities. Scale bars = 5 μm.
(TIF)

S3 Fig Generation and characterisation of SAS4-HA, SAS6-HA and SAS6-GFP transgenic lines. **A-D.** Genetic modification scheme and genotyping of the transgenic lines. **E.** SAS6-GFP microgametocytes display an abnormally shaped amorphous MTOC densely stained for SAS6-GFP. This does not seem to affect mitosis but axoneme formation and arrangement is compromised. α/β-tubulin: magenta; amine reactive groups/NHS-ester: shades of grey; centrin: blue; SAS6-GFP: yellow. Scale bars = 5 μm.
(TIF)

S4 Fig Generation and characterisation of SAS4-KO, and SAS6-KO transgenic lines. **A.** Genetic modification scheme and genotyping of the transgenic lines. **B-C. D-E.** Representative full projections of (B) non-activated and (C) 5–6 min activated microgametocytes; wild-type (1^st^ row), SAS4-KO (2^nd^ row) and SAS6-KO (3^rd^ row). α/β-tubulin: magenta; amine reactive groups/NHS-ester: shades of grey; centrin: blue; γ-tubulin: green. Boxed areas correspond to close-ups. Yellow star = mitotic spindle; yellow arrow = non-bundled microtubules; green arrow = bundled microtubules; white arrow = intranuclear body; BB = basal body. Scale bar = 2 μm.
(TIF)

S1 Table Oligonucleotides used in this study. (DOCX)

S2 Table Reagents and resources used in this study. (DOCX)

S1 Movie 3D surface topology reconstruction of the microgametocyte shown in Fig 3B. α/β tubulin: magenta; amine reactive groups/NHS-ester: grey.
(MP4)
