# Supplementary figures and images for: Expansion microscopy of Plasmodium gametocytes reveals the molecular architecture of a bipartite microtubule organisation centre coordinating mitosis with axoneme assembly
Source: PLoS Pathog. 2022 Jan 25;18(1):e1010223. doi: 10.1371/journal.ppat.1010223 (PMC8789139; doi:10.1371/journal.ppat.1010223)

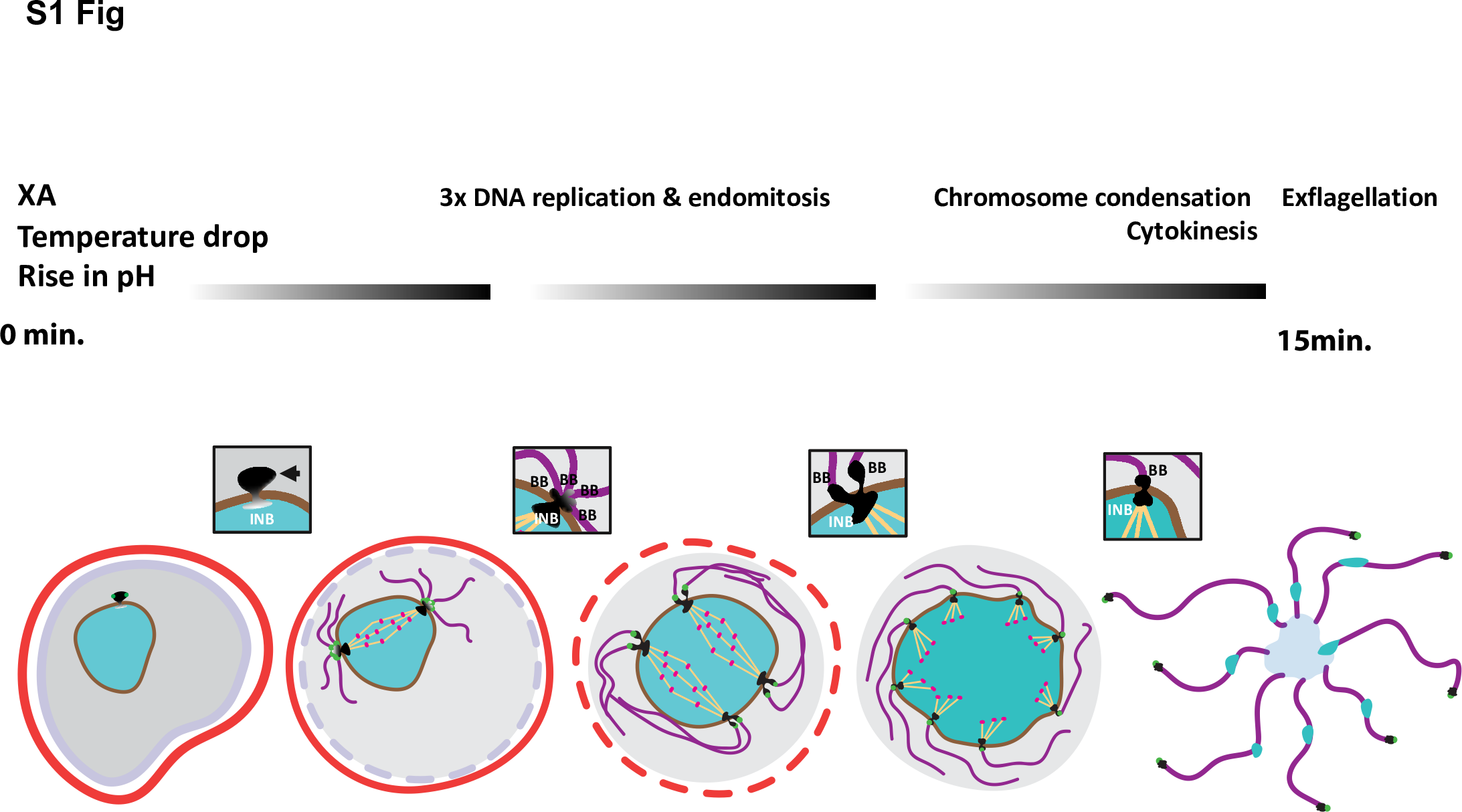

Supplement: S1 Fig — Circulating microgametocytes are arrested at a G0-like stage at the haploid level showing an amorphous MTOC (black arrow) lying on the cytoplasmic face of a nuclear pore that is physically linked to another electron dense aggregation called the intranuclear body (INB) in the nuclear face of the same pore. The molecular organisation of both structures and their link is unknown. One minute after activation, the first genome replication is completed and the spindle of mitosis I (spindle: yellow, kinetochores: pink) is observed between the two intranuclear bodies (INB). At the same time, the amorphous MTOC gives rise to 8 basal bodies (BB) which initiate nucleation of eight axonemes (magenta). By 8 minutes three successive rounds of genome replication and endomitosis have happened and at 10 minutes, full length axonemes become motile. As each basal body remains attached its corresponding intranuclear body, they drag a haploid genome that is incorporated into the exflagellating gametes. (TIF) [file ppat.1010223.s001.tif]

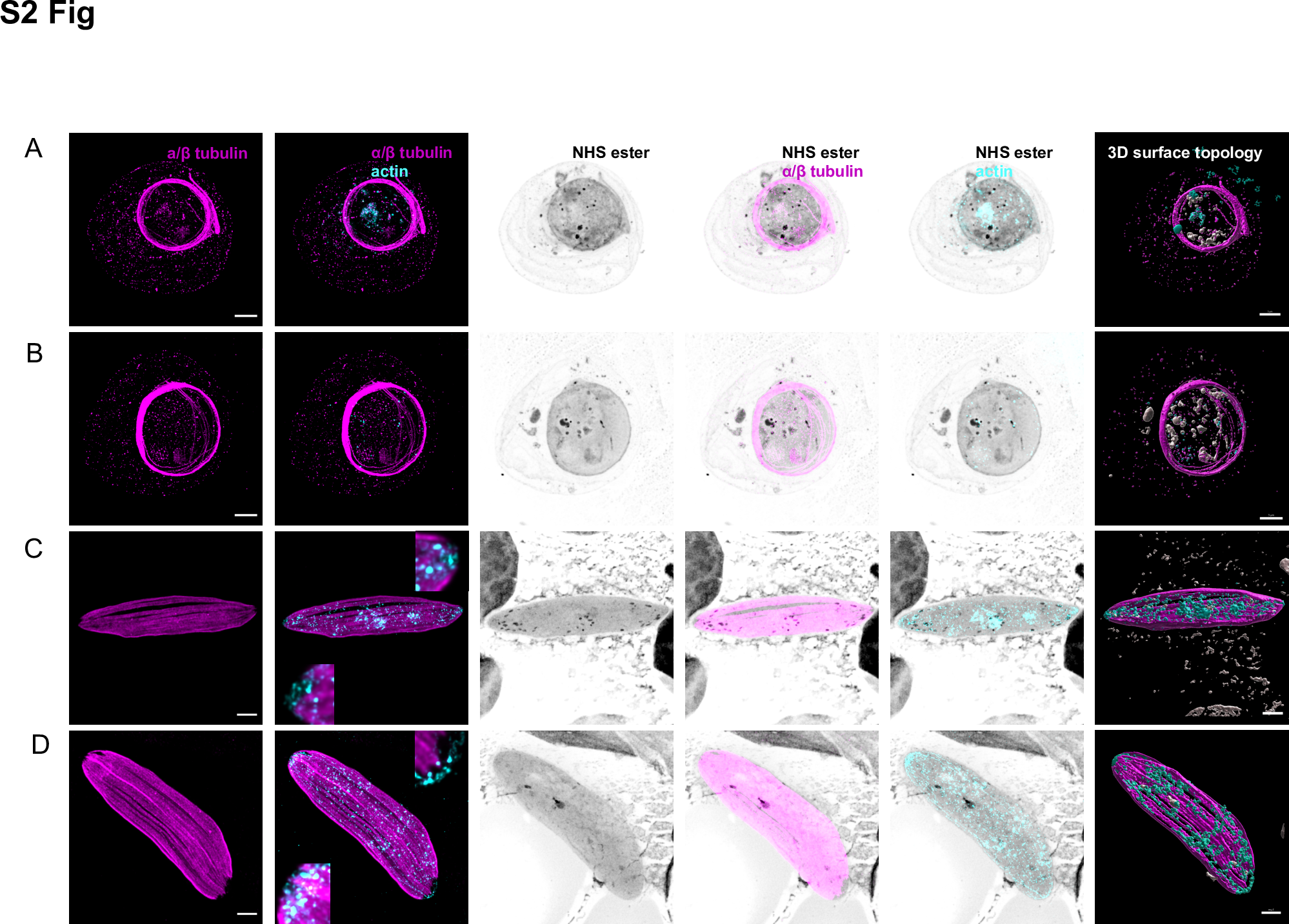

Supplement: S2 Fig — A-D. Representative full projections of P. falciparum gametocyte stages. α/β tubulin: magenta; amine reactive groups / NHS-ester: shades of grey; actin: cyan. Column 6 shows the 3D surface topology reconstruction of α/β-tubulin and actin. A and B. At early stages, a punctate distribution of actin is observed. C-D. From stage IV gametocytes, actin shows a polarised localisation, mostly concentrated at both extremities in tubulin-free areas showing a mesh-like organization. Actin also extends lengthwise alongside the subpellicular microtubules. Insets in (C and D) are close ups of both extremities. Scale bars = 5 μm. (TIF) [file ppat.1010223.s002.tif]

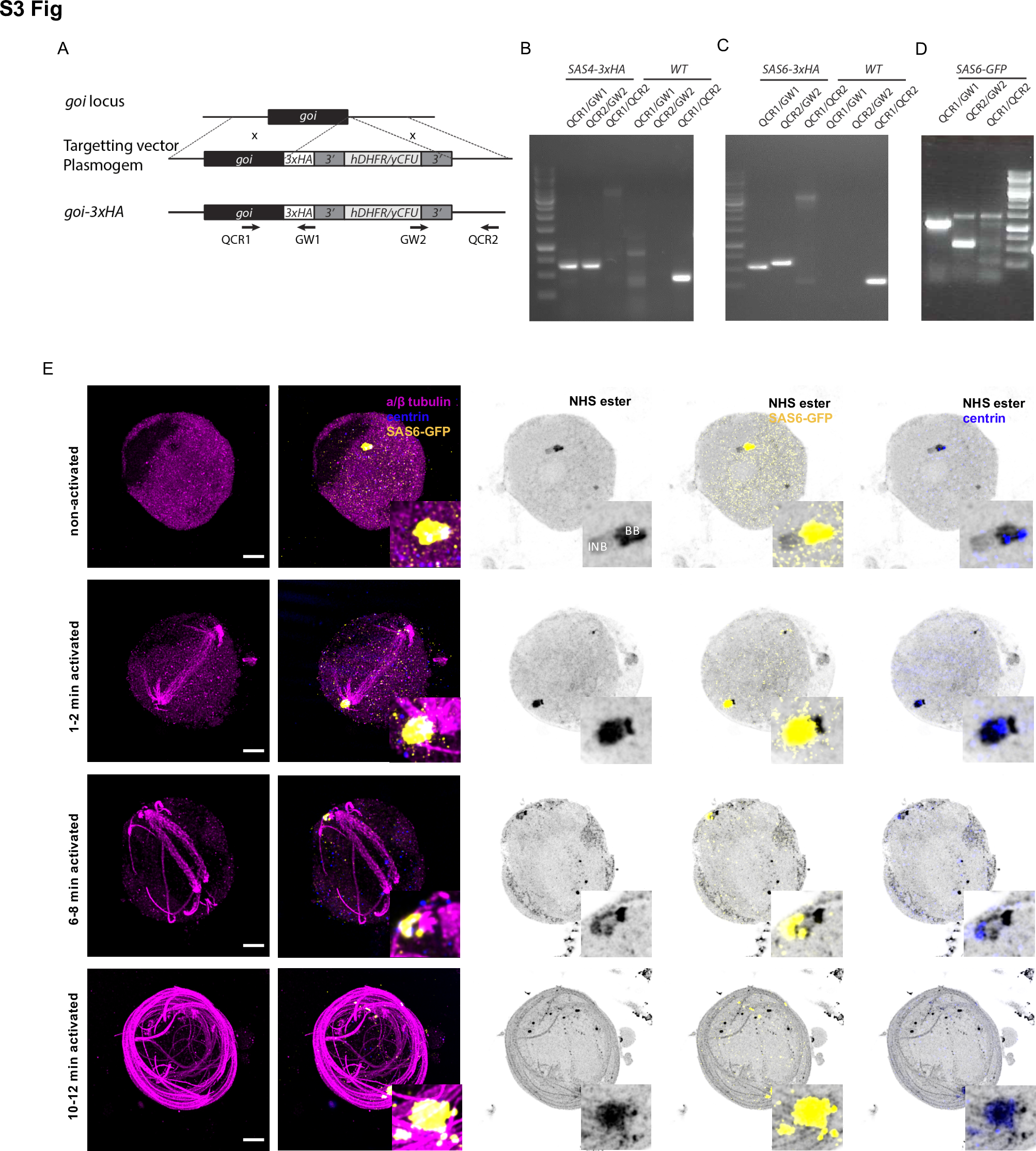

Supplement: S3 Fig — A-D. Genetic modification scheme and genotyping of the transgenic lines. E. SAS6-GFP microgametocytes display an abnormally shaped amorphous MTOC densely stained for SAS6-GFP. This does not seem to affect mitosis but axoneme formation and arrangement is compromised. α/β-tubulin: magenta; amine reactive groups/NHS-ester: shades of grey; centrin: blue; SAS6-GFP: yellow. Scale bars = 5 μm. (TIF) [file ppat.1010223.s003.tif]

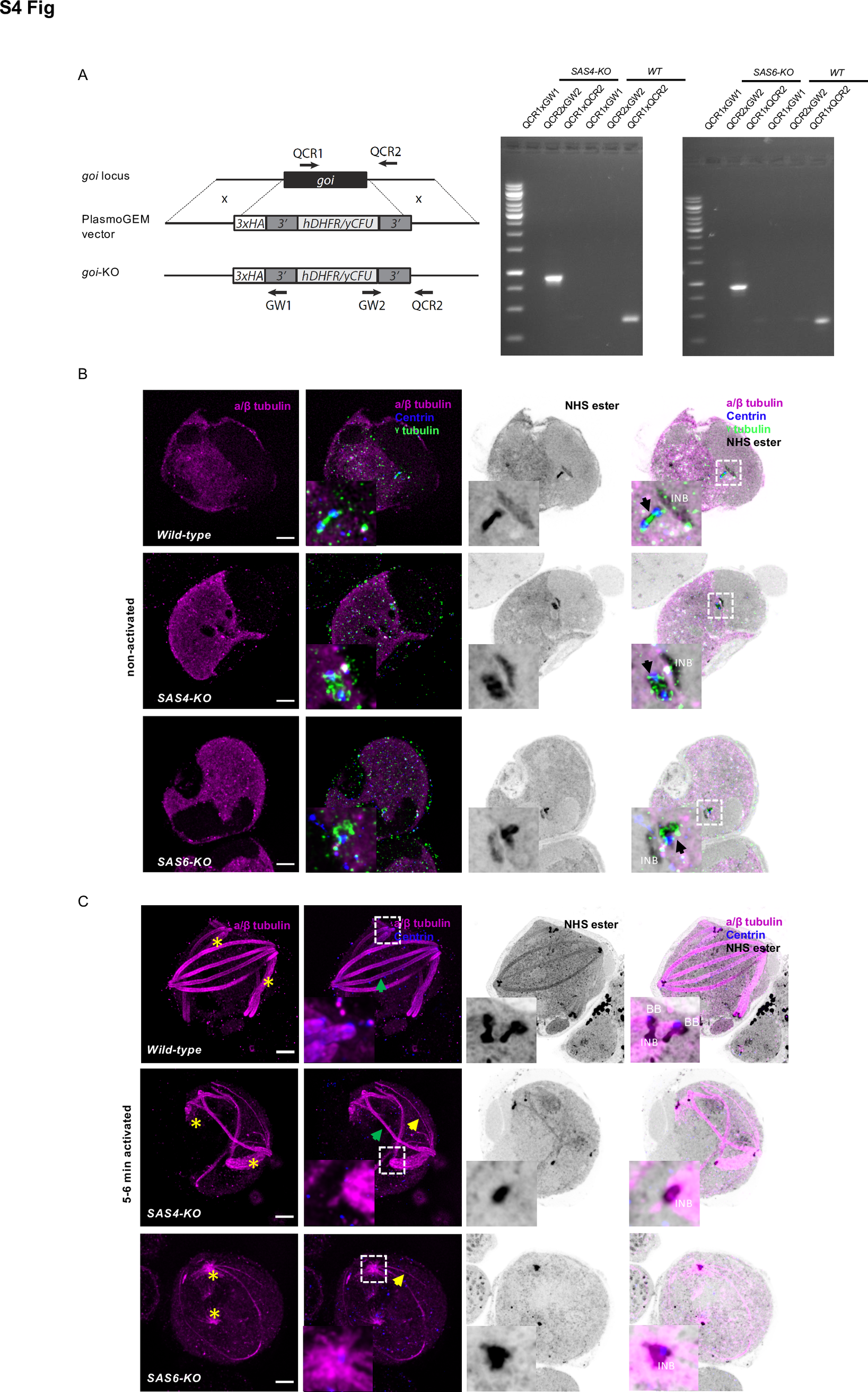

Supplement: S4 Fig — A. Genetic modification scheme and genotyping of the transgenic lines. B-C. D-E. Representative full projections of (B) non-activated and (C) 5–6 min activated microgametocytes; wild-type (1st row), SAS4-KO (2nd row) and SAS6-KO (3rd row). α/β-tubulin: magenta; amine reactive groups/NHS-ester: shades of grey; centrin: blue; γ-tubulin: green. Boxed areas correspond to close-ups. Yellow star = mitotic spindle; yellow arrow = non-bundled microtubules; green arrow = bundled microtubules; white arrow = intranuclear body; BB = basal body. Scale bar = 2 μm. (TIF) [file ppat.1010223.s004.tif]
