# Supplementary material for: Expansion microscopy of Plasmodium gametocytes reveals the molecular architecture of a bipartite microtubule organisation centre coordinating mitosis with axoneme assembly
Source: PLoS Pathog. 2022 Jan 25;18(1):e1010223. doi: 10.1371/journal.ppat.1010223 (PMC8789139; doi:10.1371/journal.ppat.1010223)
Supplement: S1 Table — (DOCX) [file ppat.1010223.s005.docx]

**S1 Table: Oligonucleotides used in this study.**

| **Primer name** | **5’ to 3’ sequence** |
| --- | --- |
| QCR1 SAS4 tag | ACAATGATGGCCACGTGCAA |
| QCR2 SAS4 tag | TCGCTAGCACCCTCAAAAAGA |
| QCR1 SAS4 KO | TCCCGACAAATTTGCGCTCT |
| QCR2 SAS4 KO | TGAGGAGCTCGAAAAGGGGA |
| QCR1 SAS6 tag | GCGGAAAAATCGATGCGCTTGGT |
| QCR2 SAS6 tag | ACGCGGATAGGGAAGTGCAGA |
| QCR1 SAS6 KO | TCGATATAATTGTGCGTCGA |
| QCR2 SAS6 KO | TGAGCGACACATAAAGACGCCA |
| GW1 | catactagccattttatgtg |
| GW2 | ctttggtgacagatactac |
