# Supplementary material for: Expansion microscopy of Plasmodium gametocytes reveals the molecular architecture of a bipartite microtubule organisation centre coordinating mitosis with axoneme assembly
Source: PLoS Pathog. 2022 Jan 25;18(1):e1010223. doi: 10.1371/journal.ppat.1010223 (PMC8789139; doi:10.1371/journal.ppat.1010223)
Supplement: S2 Table — (DOCX) [file ppat.1010223.s006.docx]

**S2 Table:** Reagents and resources used in this study.

| **Reagent type or resource** | **Designation** | **Source or reference** | **Identifiers** | **Additional information** |
| --- | --- | --- | --- | --- |
| Cell line  *P. falciparum* | iGP2 | [1] |  |  |
| Cell line  *P. berghei* | ANKA 2.34 | [2] |  |  |
| Cell line  *P. berghei* | GEX1-HA | [3] |  |  |
| Cell line  *P. berghei* | SAS4-HA | This study |  |  |
| Cell line  *P. berghei* | SAS6-HA | This study |  |  |
| Cell line  *P. berghei* | CDPK4-KO | [4] |  |  |
| Cell line  *P. berghei* | SRPK1-KO | [5] |  |  |
| Cell line  *P. berghei* | SAS4-KO | This study |  |  |
| Cell line  *P. berghei* | SAS6-KO | This study |  |  |
| Antibody | Centrin mouse (20H5) | Merck Millipore | 04-1624 | U-ExM: 1:500 |
| Antibody | α-tubulin  Guinea pig | Unige antibody platform | AA345 | U-ExM: 1:250 |
| Antibody | β-tubulin  Guinea pig | Unige antibody platform | AA344 | U-ExM: 1:250 |
| Antibody | γ-tubulin  Rabbit | Sigma | T5192 | U-ExM: 1:500 |
| Antibody | HA  Rat (3F10) | Roche | 11815016001 | U-ExM  1:250 |
| Antibody | Actin  Mouse | [6] |  | U-ExM  1:3 |
| Antibody | anti-mouse Alexa 488 | Invitrogen | A11001 | U-ExM: 1:400 |
| Antibody | anti-guinea pig 488 | Invitrogen | A11073 | U-ExM: 1:400 |
| Antibody | anti-rat  488 | Invitrogen | A11006 | U-ExM: 1:400 |
| Antibody | anti-rabbit Alexa 405 | Invitrogen | A31556 | U-ExM: 1:400 |
| Antibody | anti-mouse Alexa 405 | Invitrogen | A31553 | U-ExM: 1:400 |
| Antibody | anti-guinea pig Alexa 647, | Invitrogen | A21450 | U-ExM: 1:400 |
| Chemical | Atto 594 NHS-ester | Merck | 08741 | 10 μg/mL |
| Chemical | Hoechst 33342 | Invitrogen | H3570 | 5:1000 |

**References**

1. Boltryk SD, Passecker A, Alder A, van de Vegte-Bolmer M, Sauerwein RW, Brancucci NMB, et al. CRISPR/Cas9-engineered inducible gametocyte producer lines as a novel tool for basic and applied research on *Plasmodium falciparum* malaria transmission stages. In press Nature communications. 2020:2020.10.05.326868. doi: 10.1101/2020.10.05.326868.

2. Billker O, Dechamps S, Tewari R, Wenig G, Franke-Fayard B, Brinkmann V. Calcium and a calcium-dependent protein kinase regulate gamete formation and mosquito transmission in a malaria parasite. Cell. 2004;117(4):503-14. doi: 10.1016/s0092-8674(04)00449-0.

3. Ning J, Otto TD, Pfander C, Schwach F, Brochet M, Bushell E, et al. Comparative genomics in *Chlamydomonas* and *Plasmodium* identifies an ancient nuclear envelope protein family essential for sexual reproduction in protists, fungi, plants, and vertebrates. Genes Dev. 2013;27(10):1198-215. doi: 10.1101/gad.212746.112.

4. Fang H, Klages N, Pardo M, Yu L, Choudhary J, Brochet M. Multiple short windows of CDPK4 activity regulate distinct cell cycle events during *Plasmodium* gametogenesis. eLife. 2017;6:e26524. doi: 10.7554/eLife.26524.

5. Tewari R, Straschil U, Bateman A, Bohme U, Cherevach I, Gong P, et al. The systematic functional analysis of *Plasmodium* protein kinases identifies essential regulators of mosquito transmission. Cell Host Microbe. 2010;8(4):377-87. doi: 10.1016/j.chom.2010.09.006.

6. Herm-Gotz A, Weiss S, Stratmann R, Fujita-Becker S, Ruff C, Meyhofer E, et al. *Toxoplasma gondii* myosin A and its light chain: a fast, single-headed, plus-end-directed motor. EMBO J. 2002;21(9):2149-58.
