# Supplementary material for: Expansion microscopy of Plasmodium gametocytes reveals the molecular architecture of a bipartite microtubule organisation centre coordinating mitosis with axoneme assembly
Source: PLoS Pathog. 2022 Jan 25;18(1):e1010223. doi: 10.1371/journal.ppat.1010223 (PMC8789139; doi:10.1371/journal.ppat.1010223)
Supplement: Attachment — Submitted filename: 119068_1_rebuttal_2219349_r299xx.pdf [file ppat.1010223.s008.pdf]

Reviewer #1 (Evidence, reproducibility and clarity (Required)):

The study by Rashpa and Brochet focuses on the detailed study of the process of gametogenesis in Plasmodium, with a focus on the structural changes accompanying flagellar and basal body formation. More specifically, the study hones in basal body and nuclear MTOC genesis during macro and microgametes formation in Plasmodium using an inducible gametocyte producing cell line. The authors use bulk proteome labeling, ultrastructural expansion microscopy, PanExM and labeling of multiple specific components of MTOCs, to tackle their kinetics and structural dynamics during gametogenesis and "exflagellation" achieving unprecedented temporal and spatial resolution.
I have only identified minor issues that I reckon could be readily addressed by the authors.

Minor comments:

Line 67: You refer to the intranuclear body, and specify that this most likely corresponds to the "centriolar plaque." Work by Simon and colleagues, which you cite and refer to later in the manuscript has shown that the centriolar plaque is indeed extranuclear (which you specify later in the manuscript in Lines 87-88). Please rephrase for consistency.

We thank reviewer 1 for this comment. We have recently discussed terminology with Julien Guizetti and other colleagues and have opted to use the term intra nuclear body in gametocytes to avoid any confusion. This terminology has been used in early EM studies. We opted to avoid the term spindle pole as it has frequently been referred to as the spindle pole body, a terminology that describes a yeast MTOC. Similarly, the term centriolar plaque was mainly used to describe the mitotic MTOC in *Plasmodium* asexual blood stages and is different from the one we describe here.

Line 80: The wording on sentence starting with "At the onset of exflagellation.. " is confusing. Please rephrase for clarity

We have rephrased this sentence.

The transition between lines 83 and 84 is quite abrupt. I suggest a connecting phrase be added between these two seemingly disconnected paragraphs to smooth the transition, and aid the reading.

We have rephrased this section.

Line 90: Here you refer to the centriolar plaque again, but specify that it does not contain centrioles, and therefore you'll refer to it as the "acentriolar plaque." I suggest you move this to earlier on in the introduction to aid the reading.

We have decided to use the term intranuclear body rather than acentriolar plaque.

Line 104 and Line 106: While UExM and PanExM are widely popular these days, please provide the

full name the first time you refer to these techniques in the manuscript, for clarity.

We are now providing the full names.

Line 119: please specify that iGP2 refers to an "inducible gametocyte producing" cell line for clarity. Briefly describe what the comparative advantages of using these cell lines are, i.e. what they allow for, and how they work, for non-expert readers.

We are now indicating that the iGP2 line is “an inducible gametocyte producing line that allows for the routine production of large numbers of viable P. falciparum gametocytes in vitro”. More details can also be found in the method section.

Line 136 specifies that "a low NHS ester density" is observable adjacent to the nucleus in Figures 1E and F. This region is not immediately visible. Please aid the reader by highlighting it in your figure and referring to it in the figure legend.

We have added arrow heads to highlight this region

Lines 141-142: As presented, this suggestion seems purely speculative. Either remove this sentence or provide evidence that the NHS-densely labeled region on the nuclear side corresponds to the "acentriolar" plaque.

We agree with reviewer 1 this is highly speculative. We have removed the comparison with the centriolar plaque of the asexual blood stages.

Lines 148-154: It is unclear to me what the significance of labeling actin is, in the context of the manuscript's focus. Please clarify. In addition, the actin labeling pattern is described for stages IV and V, but the labeling patterns of earlier stages (shown and briefly mentioned in the figure's legend) are not. Please describe the data shown. Is the lack of staining in Stages II-III expected? Please clarify.

We agree with reviewer 1 that labelling actin is not of critical importance in the context of this manuscript and Figure 2 is now moved to the supplementary materials.

For its mechanistic insight, the manuscript relies heavily on the statement that densely labeled structures by NHS-esters are basal bodies or extranuclear acentriolar plaques. The 3D surface topology provided in Figure 3 and MovieS1, while illustrative, do not fully resolve the structures. The evidence that these densely NHS-ester labeled proteinaceous structures are most likely basal bodies and/or the acentriolar plaques is shown in Figure S1E. I suggest you move this part of the figure to the main Figure 3. It is mentioned in line 164 that NHS-ester also likely labels the kinetochores at the end of the mitotic spindle. This is a speculative statement; either remove it or evaluate this using anti-CenH3 antibodies to label centromeres.

We agree with reviewer 1 that at this stage, no strong evidence is given to support that the NHS ester dense staining corresponds to the basal body. In order to avoid mixing parasite species between the figures, we have not moved Fig S1E to the main Fig. 3. However, we have changed the main text as follow: “On the cytoplasmic face of each intranuclear body, four NHS-ester dense structures were associated with orthogonally arranged short axonemes and likely include the basal bodies”. The SAS6-HA and SAS4-HA labelling are subsequently shown in Figure 6.

We also concur with reviewer 1 that it was premature to label these NHS ester dense structures as kinetochores. We are now citing our recent preprint (https://doi.org/10.1101/2021.11.03.466924) on the characterization of the apicomplexan kinetochores that supports this suggestion.

According to your figure legend, Figure 7E-H are mislabeled; SAS4 is written instead of SAS6.

We thank reviewer 1 for spotting this mistake that we have now corrected.

Reviewer #1 (Significance (Required)):

The manuscript is very clearly written; it is concise, easy and enjoyable to read. The problem is clearly identified, and so are the experimental approaches selected to tackle the proposed questions. The state of the art in the field is clearly conveyed and summarized. Overall, the study provides valuable insight into the biology of MTOCs during gametogenesis, shedding light onto an ill-understood process central to the viability and transmission of the malaria parasite. The data are of supreme quality, and the discussion thought provoking. I very much enjoyed reading and reviewing this manuscript.

My expertise is on cell division and MTOC biology in Toxoplasma gondii

We thank reviewer 1 for their constructive and positive comments.

Reviewer #2 (Evidence, reproducibility and clarity (Required)):

Summary:

This paper uses a new microscopy technique (ultrastructure expansion microscopy, U-ExM) to investigate cell biology of the sexual stages of malaria parasites. In the first part they focus on the human parasite Plasmodium falciparum and investigate gametocytogenesis, which uniquely in this species takes 14 days and involve major changes in cell structure. They were able to determine the 3D structures of the cytoskeleton, improving resolution compared to previous studies. They then used the same technique to study gametogenesis in P. falciparum and P. berghei, the latter being the main subject of the study. Male gametogenesis is a rapid process where within ~10 minutes there are three rounds of mitosis and simultaneously the assembly and activation of eight axonemes resulting in the formation of eight flagellar gametes each with a haploid genome. The authors focus on the so called microtubule organizing center (MTOC) which coordinates these processes. They follow the dynamics of the MTOC during gametogenesis and they describe in detail the organization of the MTOC and localize several proteins to different subdomains. Double labeling using immunofluorescence assay on expanded cells combined with NHS-ester labeling of protein rich organelles offers an unprecedented detailed description of the dynamic MTOC during gametogenesis. These new findings localizes proteins of importance for mitosis and axoneme assembly. Furthermore, the phenotypic analysis of two kinases previously known to play a role in male gametogenesis now clarifies in detail their function and suggest that they are required for proper formation of the basal bodies required both for correct assembly of the spindle and the axonemes.

Major comments:

The manuscript is convincing and the experimental data supporting the conclusions. The manuscript is complete and no additional experiments are required. However as explained below in some figures a magnification of smaller areas of the cell would help understanding specific features.

The method section includes detailed description of the methods used allowing the experiments to be repeated.
The data in this paper is based on microscopy images of single cells. However taken as a whole the findings are replicated in the different parts. Statistical analysis is not required for such a study.

Minor comments:

References pertinent to the subject are included, with both recent and older literature cited. In a few cases (see below) the references should be reviewed by the authors.
I find the paper in certain places difficult to follow and therefore suggest the following minor changes.

Abstract: please state that P. berghei is used in the study of gametogenesis

We thank reviewer 2 for this suggestion that will prevent any confusion.

As the main part of the study on gametogenesis is carried out on P. berghei a few sentences on whether the findings are equally valid for P. falciparum should be included in the Discussion.

Again, we agree with reviewer 2 that it is important to clearly separate the two species. As suggested, we have modified the discussion (lines 371-374).

Introduction: A brief description of the U-ExM and NHS-ester labeling techniques would help the reader understand the experiments.

We thank reviewer 2 for this comment. We are now briefly detailing this technique (lines 120-128)

Line 55. While the authors refer to osmiophilic bodies in females, males have also been found to contain similar organelles (PMID:25262869).

We thank reviewer 2 for pointing to this important reference we are now citing in the section describing *P. berghei* gametocytes (lines 180-182).

Lines 68-76. Insert reference for this section.

We now cite an excellent review by Pr Sinden (doi.10.1016/j.mib.2010.05.016) for this section.

Line 77. Reference 11 does not refer to Plasmodium

We thank reviewer 2 for spotting this mistake that we have corrected.

Line 78. Insert reference

We are now referring to the review by Pr Sinden (doi.10.1016/j.mib.2010.05.016).

Line 86. The authors suggest changing the name "centriolar plaque" to "acentriolar plaque" on line 90, and I suggest inserting on line 86 "the until now known as centriolar plaque" to prepare the readers for the name change.

As explained in our response to reviewer 1, we have decided not to use the term acentriolar plaque to avoid any confusion.

Line 96. Reference 15 does not describe the basal body.

We have corrected the wrong reference.

In general I find the Figure legends to lack sufficient description of the pictures to make them easy to understand. Fig. 1A, there are big gray areas close to the parasite, can the authors suggest what these are? There are no labeling suggestive of OBs in Fig. 1C-E, is there a reason for this? Fig. 1E and F, the authors refer to a low density labelling region near the nucleus, should be indicated in the picture with an arrow. It would be helpful if the nuclear periphery was indicated with a dotted line.

We do not know what the big grey areas are. We have observed them in 3 out of 5 cells at this stage. They appear to be in the host red blood cell (please see enlarged 3D surface topology reconstruction below). We now mention these areas in the main text.

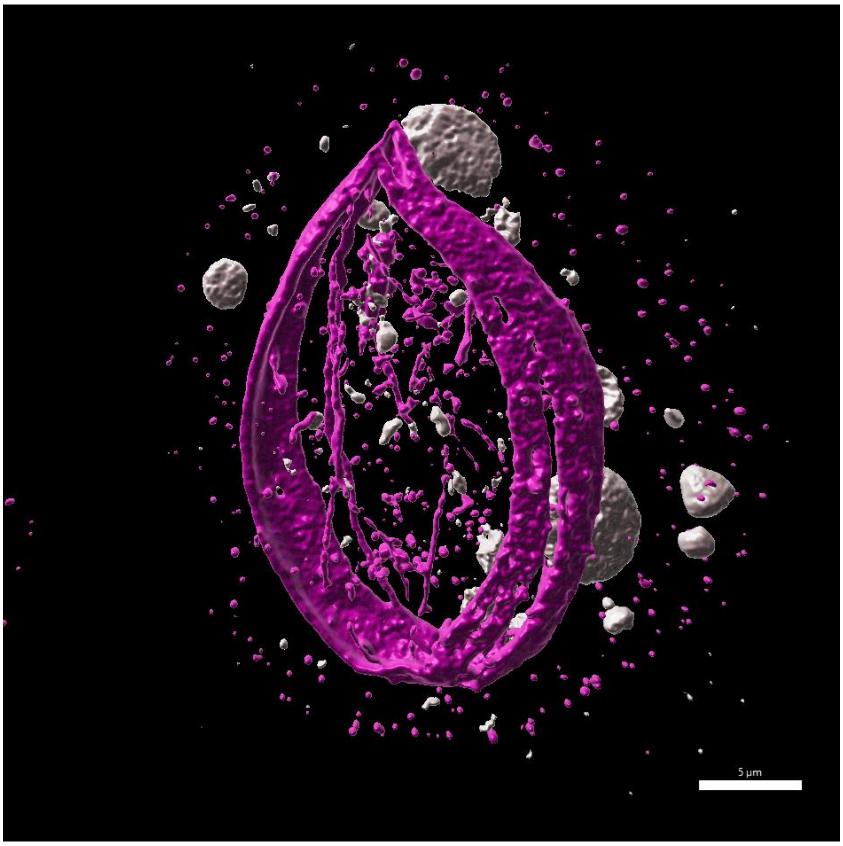

We are now indicating representative osmiophilic bodies with white arrow heads in macrogametocytes. As explained above we also indicate that male osmiophilic bodies are not stained under our experimental conditions. Our unpublished results indicate that different flavors of NHS ester dyes label ookinete micronemes differently. Further analysis will be required to identify dyes that may label osmiophilic bodies in microgametocytes. We also highlight the nuclear periphery in Fig. 2B as suggested.

Line 134-135, Fig. 1E, F. Sutures connecting the MTs are mentioned. Could these be indicated in the figures?

The suture between IMC plates are now highlighted in Fig. 1E and 1F.

Line 153 and Fig. 2. The statement that actin labelling extends alongside the microtubules lacks clarity. Does the author means that there is co-localization of the actin with the MTs? From the Fig. it is not easy to say if this is the case as the MTs are very close to each other.

We do not have the resolution to exactly localize actin with respect to the subpellicular microtubules. This is why we used a general term to describe actin localization.

Line 160 and Fig. 3A and B. It is very difficult to see the surrounding erythrocyte due to low contrast, this should be indicated in the legend.

We agree with reviewer 2 and are now indicating a lighter NHS-ester staining in late gametocyte stages.

Line 165 onwards Fig. 3B. The features discussed are very difficult to discern in the pictures, especially that there is no overlap between spindle and APs. I suggest including higher magnifications to make these points clear and indicating the nuclear periphery with a dotted line.

We have enlarged this figure and highlighted the nuclear membrane. The structure is also visible in movie S1.

Line 177. In male gametocytes it has been reported that a double membrane is present, although no subpellicular MTs have been seen (for example https://pberghei.nl/wp-content/uploads/2021/04/Pictures-Chapter-Morphology-of-Pberghei-EM_2.pdf refers to a third membrane). I suggest the sentence be modified accordingly.

We indeed indicate that neither subpellicular microtubules nor IMC plates are detected in *P. berghei* gametocytes. As NHS-ester seems to highlight membrane negatively it is difficult to comment on the presence of this third membrane. It is possible that future analyses using membrane staining as described in doi 10.1101/2021.09.25.461816 will allow to characterise these structures.

Line 184-185. No OBs were seen in males using this NHS-ester labeling, do the authors have any explanation for this?

As explained above we now indicate that male osmiophilic bodies are not stained under our experimental conditions. Our unpublished results indicate that different flavors of NHS ester dyes label ookinete micronemes differently. Further analysis will be required to identify dyes that may label osmiophilic bodies in microgametocytes

Line 193-194 Fig. 5E. The Hoechst staining is very difficult to see, a higher magnification should be provided.

Due to the size of the figure it is difficult to show a higher magnification. Instead, we have switched the Hoechst color from orange to green that, in our view, highlights better the staining.

Fig. 5F. This figure is difficult to understand. The legend should explain in more detail the figure. If I understand correctly each number indicates a mitotic spindle linked to the basal body of the corresponding axoneme. However, box 7 does not have an axoneme attached to it, and box 4 doesn't show a spindle. Are these found in a different z-section? This should be clarified in the legend. In addition labeling the top row "7" and the bottom row "4" would help for understanding the various parts of the figure. What are the strong staining dots in box 4, especially visible in the right-most sections (bottom row). The proteinaceous filament mentioned in line 201 should be indicated in the figure. Possibly giving each section a different letter (a, b etc) will help the reader in following the reasoning.

We are now indicating precisely which sections are shown in the central panel and in the close ups. We hope this makes it easier to understand each panel. We now indicate the proteinaceous filaments with arrows.

Line 267. Have the authors looked at whether basal bodies are formed?

This is an interesting question! In microgametocytes, the absence of SAS4 or SAS6 leads to the progressive loss of the NHS-ester dense basal bodies (new figure 8). This NHS-ester dens region is till observed in the absence of SRPK1 suggesting that a structure related to the basal body is formed. This is further supported by the nucleation of microtubules. However, details on the molecular organization of the basal body in absence of SRPK1 would require labelling known or not yet known markers of the basal body.

Figure 10 provides a schematic summary of the findings and it is highly recommended that it is included in the final manuscript to help the non-specialist reader understand the complex and dynamic processes during microgametogenesis.

We thank reviewer 2 for this suggestion; we have kept and improved the figure.

Line 331. Insert a reference for this statement.

We have added a reference describing the low number of centriolar proteins in *Plasmodium*.

Reviewer #2 (Significance (Required)):

This is an exciting paper significantly furthering our understanding male gametogenesis in malaria parasites. The new microscopy technique that has been used allows a detailed understanding of the hitherto elusive MTOC of this stage of the malaria parasite due to the improved resolution combined with immunofluorescence to localize proteins of interest.

Male gametogenesis has been studied using molecular genetics and microscopy methods previously and the study here uses this knowledge to reach new understanding of this process. For example several of the proteins that are investigated in this study have been identified as having important roles in male gametogenesis and are duly referenced.

This paper will be a landmark for those researchers interested in cell biology of the malaria parasite and specifically male gametogenesis. It will also be of interest to those studying mitosis and/or axoneme formation in other organisms and the evolution of these processes. Additionally, understanding of this essential stage in the life cycle may assist in developing drugs for blocking of malaria transmission through the mosquito.

My expertise is within malaria parasite molecular genetics and cell biology with an emphasis on the mosquito stages. I have no direct experience of the U-ExM method used here.

We also thank reviewer 2 for their very detailed, constructive and supportive comments which certainly helped improve the manuscript.

Reviewer #3 (Evidence, reproducibility and clarity (Required)):

In this manuscript, Rashpa and Brochet make attempts to recapitulate cellular events during gametogenesis of malaria parasite Plasmodium. Expansion microscopy has become a useful tool to visualize and investigate cellular structures at high resolution. In this study, Ultra structural (U-ExM) and Pan- Expansion (P-ExM) microscopy is used to characterize developmental processes during macro- and microgametogenesis. These studies suggest that MTOC organizes axoneme formation during mitosis and the MTOC transforms to two tetrads of basal bodies (BBs), which are linked by mitotic spindles. BBs seemed to emerge from a "deuterosome-like" structure. A bipartite MTOC has been proposed to regulate the formation of axonemes during mitosis. In addition, two previously described mutants of P. berghei in which kinases CDPK4 and SRPK1 are disrupted were used to establish the involvement of these kinases in MTOC organization.

Previous studies performed using electron microscopy as well high-resolution fluorescence microscopy have unraveled several of the above-mentioned events. However, given the dynamic nature of some of the cellular processes involved in gametogenesis several events remain poorly defined. While present studies provide improved "resolution" for some of the key processes, they only provide an incremental advancement to our understanding of gametogenesis. Here are some issues that need to be addressed:

a. While Introduction provides a detailed account of the current understanding of gametogenesis, it will be nice to provide a schematic illustrating various steps involved in this process. It will help define the problem and will especially be useful for researchers from other areas.

We thank reviewer 3 for this suggestion. We have now added a schematic illustrating microgametogenesis in Figure S1.

b. It will be nice to track down IMC during gametogenesis using an IMC marker.

We agree with reviewer 3 that it would be nice to track down IMC assembly and disassembly, an exciting line of research we are currently investigating. We however believe that such analysis is not related to the main focus of this manuscript that aims at better describing the MTOC coordinating mitosis and axoneme formation.

c. Actin polymerization-depolymerization inhibitors should be used to study cytoskeleton and microtubule organization and organelle assembly.

This is also a very interesting suggestion and such inhibitors have already been tested on the actin cytoskeleton of *P. falciparum* microgametocytes in an excellent paper for the group of Pr Tilley (doi.org/10.1111/cmi.12359). In this article, the authors nicely show that the actin-depolymerizing agent, cytochalasin D, depletes actin from the end of the gametocytes, whereas the actin-stabilizing compound, jasplakinolide, induces formation of large bundles and prevents late-stage disassembly of the actin cytoskeleton.

Although we are not sure which organelle reviewer 3 is referring to, it was also shown that long-term treatments with cytochalasin D or jasplakinolide were associated with disruption of the normal mitochondrial organization and decreased gametocyte viability. We currently have not tested markers of the mitochondrion with U-ExM but this is also a line of research we are currently investigating. We feel that addressing the exciting questions proposed by reviewer 3 would not fit directly with the scope of this manuscript and as suggested by reviewer 1 we have moved the imaging of the actin cytoskeleton to the supplementary material.

d. Figure 4: The resolution is poor, therefore, another set of images are needed.

We believe that the apparent poor resolution was due to the color choice. We have changed the yellow to cyan which appears better to us.

e. Figure 9: Even though axoneme formation is delayed, it is mentioned that 3-4 axonemes are formed in CDPK4-KO parasites. While this may be true, these axonemes seem to be stunted. In the case of SRPK1-KO, at this stage axonemal MTs seemed to have elongated but were not organized properly. The possible reasons for these differences should be discussed.

We thank reviewer 3 for this interesting observation. In well studied models, the formation of axoneme is a highly regulated process. As the cytoplasmic assembly of the *Plasmodium* axoneme appears to differ significantly from known models together with the fact that most canonical regulators of axoneme assembly are missing or too divergent to be detected in *Plasmodium*, it is difficult to speculate why the deletions of *cdpk4* or *srpk1* lead to different defects in axoneme assembly. However, the new results we have included show that deletion of *sas4* or *sas6* also lead to a spectrum of defects. In light of these new results and in combination with our response to comment f, we now discuss these important differences.

f. Do CDPK4 and SRPK1 work in the same pathway or act independently? It will be good to study the dynamic localization of these kinases during gametogenesis. In the absence of these and other direct evidence related to CDPK4 and SRPK1, I feel the schematic in Figure 10 is too hypothetical.

The role of CDPK4 during microgametogenesis has attracted significant research attention and at least three roles have been described for this kinase during microgametogenesis. This includes the initiation of DNA replication, the formation of mitotic spindle and the formation of axonemes. We and other groups have relatively well studied the requirement for CDPK4 in the initiation of DNA replication but its role in the formation of mitotic spindle and axonemes remained elusive. By implementing U-ExM, we are now able to understand that this requirement happens very early as, in the absence of *cdpk4*, the MTOC homeostasis is blocked or strongly impaired. This is what we indicate in Fig 10. Until now the exact cellular requirements for SRPK1 was also unclear and we could show here that it is important for the correct molecular organization of the deuterosome-like structure during gametocytogenesis and of the resulting MTOC during microgametogenesis as shown in Fig 9, which we depict in Fig. 10. As stated by reviewer 2, we do believe that our model is important to summarise this work and describes what is observed in this manuscript. In the model shown in Fig. 10, we describe the general requirements of SRPK1 and CDPK4 that we have observed in our analyses. We do believe that these functional observations do not require to know the localization of these two enzymes.

We would like thank reviewer 3 for asking an exciting question about the functional relationship between CDPK4 and SRPK1 which we did not discuss previously. We are now discussing this aspect based on our previous phosphoproteome analyses (PMID29141230) which suggested that SRPK1 and CDPK4 regulate similar biological processes linked to the microtubule skeleton in gametocytes. In these phosphoproteome analyses, CDPK4-KO and SRPK1-KO samples were enriched for microtubule cytoskeleton in negatively disrupted proteins in CDPK4-KO and in both positively and negatively disrupted proteins in SRPK1-KO. Specifically, in the case of CDPK4 the phosphosites were upregulated in WT and unregulated in the KO after activation; while for SRPK1 the trend was reversed. Notably, the specific phosphosites that were disrupted did not overlap between mutants, suggesting that the two protein kinases have different sets of substrates. However, in activated gametocytes, we found that SRPK1 is phosphorylated in a CDPK4 dependent manner suggesting a direct interplay between the two kinases. We are currently further investigating this complex issue and do believe that such analysis is not required to support the main conclusions of this study.

g. The authors do not cite and discuss recent papers: Wall et al., Scientific Reports 2018 report mutants which exhibit defects in axoneme assembly and nuclear pole formation during gametogenesis and are pertinent to present studies. A recent preprint (Yahiya et al BioRxiV 2021) also reports the investigation of microgametogenesis by using 4D-live cell imaging. It will be nice to compare present results with the ones in this study. I feel that these and other reports in which high-resolution fluorescence microscopy and/or EM provide some of the information which is as good as reported in the present work.

We would like to thank reviewer 3 to point to the preprint from Yahiya et al (BioRxiV 2021) that shows beautiful time-lapse imaging. However, this preprint was published on July 29th 2021, a week later after we submitted our manuscript on July 21st 2021 to bioarxiv. We are now citing this work in

the discussion to better highlight that U-ExM represents a complementary approach to EM or live-cell imaging.

We have also included the possible role of ubiquitination in the homeostasis of the microgametocyte MTOC and now cite the nice work from Prof Tewari (Wall et al, scientific report 2018 and Guttery et al, plos pathogens, 2012). However, we would like to stress that in both articles, the authors do not report mutants which exhibit defects in axoneme assembly.

Reviewer #3 (Significance (Required)):

Present studies shed light on key cellular events that guide gametogenesis using expansion microscopy.

Audience: Present studies will be of interest to malaria researchers.

My expertise: Cell Biology and cell signaling, malaria
